# New paleomagnetic results from Neogene to Quaternary volcanic rocks of north of the Lake Van, Eastern Turkey

**DOI:** 10.1038/s41598-023-39492-w

**Published:** 2023-07-27

**Authors:** Sercan Kayın, Turgay İşseven

**Affiliations:** 1 Department of Mining and Mine Extraction, Gümüşhane Vocational School, Gümüşhane University, 29100 Gümüşhane, Turkey; 2 Department of Geophysics, Faculty of Mines, Istanbul Technical University, 34469 Maslak, Istanbul, Turkey

**Keywords:** Geomagnetism, Palaeomagnetism, Tectonics

## Abstract

The Eastern Anatolia is an active tectonic region where the collision between the Arabian and Eurasian plates take place. Due to the subduction of Arabian plate’s oceanic lithosphere under Eurasian plate, widespread volcanism observed in large areas began in Serravallian. There is no consensus in the literature for the tectonic evolution of the region. Therefore, there are many geological and geophysical studies conducted with the intention of explaining the tectonic evolution of Eastern Anatolia by geodynamic models. Our paleomagnetism study aims to reveal the tectonic rotations in order to better understand the development of the prevailing tectonism in the region from the volcanic rocks. Paleomagnetic samples were collected from 86 sites of the Late Miocene–Pleistocene volcanic rocks located at the north of Lake Van. Isothermal remanent magnetization studies show that magnetite is the mineral responsible for magnetization in most rocks, while both magnetite and hematite are responsible for the rest of the rocks. Curie temperatures and alteration degrees of rock samples were also determined by high-temperature susceptibility (HTS) studies. In some samples, titanomagnetite component was observed in the heating phase of the HTS measurements. The absence of this component in the cooling step indicates that Ti-magnetite is transformed into magnetite by alteration. The Pleistocene volcanics show counterclockwise rotation of R ± ΔR = 13.4° ± 3.8°. The Pliocene volcanic rocks were defined in four different groups: south of Erciş Fault, north of Erciş Fault, around Muradiye and north of Van. Also, the remarkable clockwise rotation is observed in the north of Van and near Muradiye R ± ΔR = 24.4° ± 17.0° and R ± ΔR = 6.9° ± 9.4°, respectively. In addition, counterclockwise rotation (R ± ΔR = 14.5° ± 6.1°) is obtained in the southern part of the Erciş Fault, while there is no significant rotation (R ± ΔR = 0.6° ± 7.4°) on the northern side. Late Miocene volcanic rocks show no significant rotation either (R ± ΔR = 1.8° ± 13.7°). Our new paleomagnetic results indicate that the left-lateral strike-slip Çakırbey Fault, located to the east of the Erciş fault and extending roughly in the northeast–southwest direction, may be active.

## Introduction

The collision between Arabia and Eurasia is considered to be the beginning of the neotectonic regime in and around Turkey by some researchers^1,2^. Şengör^2^ stated that the northward movement of the Arabian plate and westward movement of the Anatolian plate formed four neotectonic regions: The East Anatolian Contractional Province, the North Anatolian Province, the Central Anatolian Ova Province and the West Anatolian Extensional Province. In the literature, there are different views regarding the age of this collision, including the late Cretaceous^3–5^, the late Eocene–Oligocene^6–8^, and the Miocene^9–16^.

**Figure 1:**
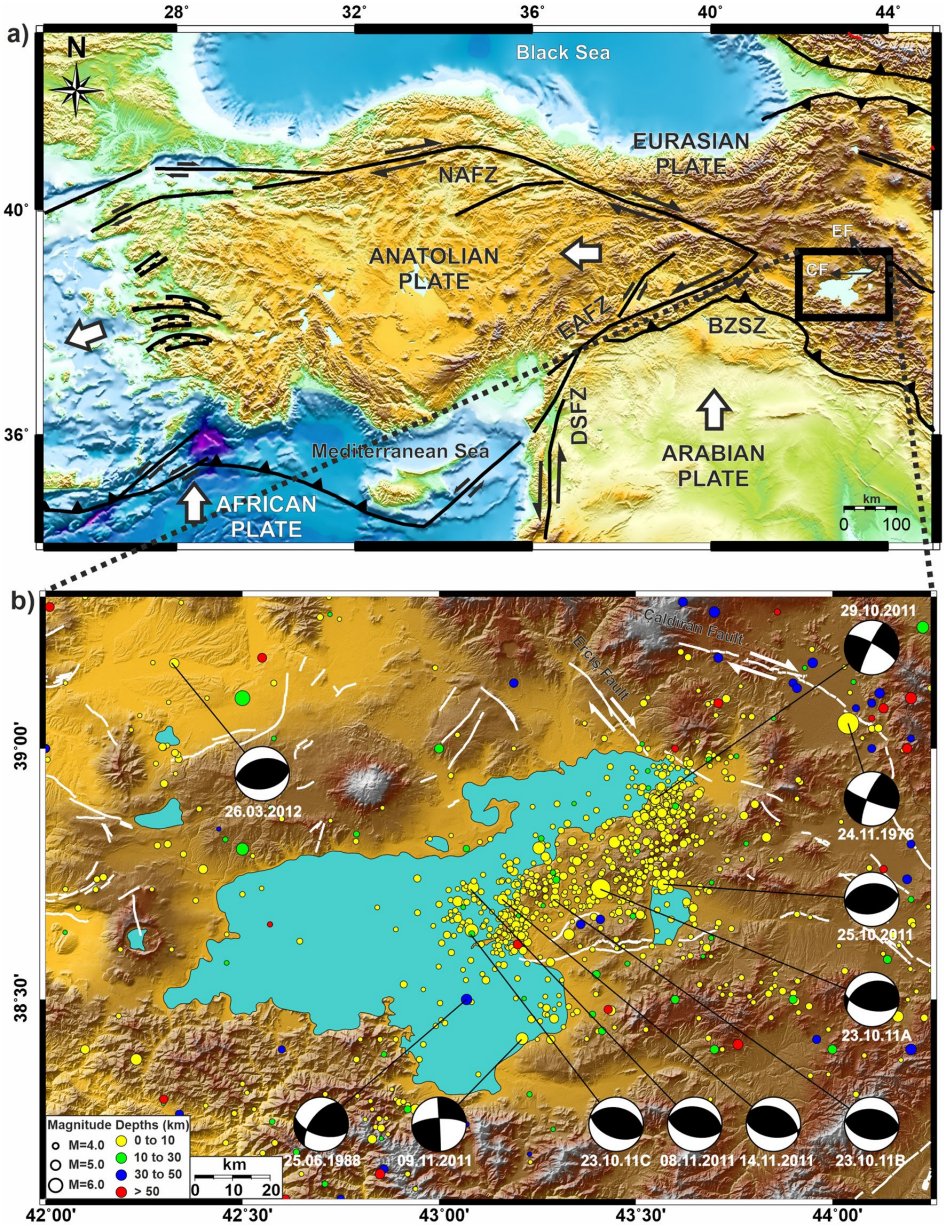
(**a**) Tectonic map of the Anatolian, Eurasian and Arabian plates, NAFZ: North Anatolian Fault Zone; EAFZ: East Anatolian Fault Zone; DSFZ: Dead Sea Fault Zone. (**b**) Seismicity of the Lake Van and its surroundings (M > 4.0 Earthquakes between 1900 and 2019 (earthquake epicenter data from Kandilli Observatory and Earthquake Research Institute (KOERI) and focal mechanism solutions of M > 5.8 earthquakes between 1976 and 2019 (retrieved from “Global Centroid Moment Tensor Catalog”,^82,83^). Maps were created by using the Generic Mapping Tools software, version 5.1.1. (https://www.soest.hawaii.edu/gmt/)^84^.

The collision between Arabian and Eurasian plates led to a large plateau formation reaching about 2 km in elevation in Eastern Anatolia^1^. During Miocene, the Anatolian plate, between the North Anatolian Fault Zone (NAFZ) and the East Anatolian Fault Zone (EAFZ), started to move westward (Fig. 1a)^10,17–19^. Due to the compressional tectonic regime in the Eastern Anatolia, east–west trending folds, thrusts, and strike-slip fault systems are formed, ongoing activity of which still can be observed from focal mechanism solutions of the earthquakes in the area (Fig. 1b)^1,10,12,18,20–25^.

East Anatolian Contractional Province consists ophiolitic melange and flysch^15,26^ that are overlain by Early and Late Miocene sediments. Most of these sediments are then covered by Lower-Mid Miocene lavas^13,27–29^. Many researchers argue that the volcanism that developed in Eastern Anatolia has started in the Late Miocene and continued until today^27,28,30–36^. Volcanic activity began soon after the region's uplift and produced volcanic material in many countries, including Turkey, Russia, Georgia, Azerbaijan, Armenia, and Iran. Collision-related volcanism in the Eastern Anatolia covered almost two-thirds of the region and formed volcanic products reaching up to 1 km in thickness^29^. Keskin^28^ related this volcanism and the rapid uplift of the region with the delamination of the subducted lithosphere under the Eastern Anatolia. The products of the post-Miocene volcanism are encountered throughout the entire region and among the volcanic centers: Erzurum Kars plateau (EKP)^13,27^, Nemrut Volcano^36^, Süphan Volcano^37,38^, Tendürek Volcano^39,40^, Karacadağ Volcano^41^ and Etrüsk Volcano^42–44^ have been studied in detail by many researchers.

In this paper, the results of new paleomagnetic and rock magnetic studies conducted in the north of the Lake Van, Eastern Anatolia are presented. There are only a few previous paleomagnetic studies available close to the research area. The study that was carried out on the Quaternary volcanics throughout Turkey by Sanver^45^ includes only one site close to our study area indicating a clockwise rotation. Another study Hisarlı et al.^16^ claimed that there exist at least five blocks bounded by left and right lateral strike-slip faults in the Eastern Anatolia, and noted that the Van Block (VB), rotated clockwise as a single rigid block. Gülyüz et al.^46^ studied Neogene sedimentary rocks in the southeast of Lake Van, and reported that these sedimentary rocks have a significant 25° clockwise rotations.

The objective of our study is to investigate the tectonic development and related tectonic deformations observed in the region using paleomagnetic samples collected at 86 sites in the north of the Lake Van. In this context, we sampled Late Miocene, Pliocene and Pleistocene volcanic rocks whose ages and rock types are known from the literature^40,43,44,47^.

## Geological setting and paleomagnetic sampling

The geological evolution of the region is defined in four different periods^20^. The first period is designated by the generation of the Paleozoic–lower Mesozoic metamorphic rocks^48–54^. The second period is represented by the Upper Cretaceous ophiolitic accretionary complex^50,55–59^. The third period is depicted as sedimentary rocks deposited between Late Cretaceous and Miocene^1,60,61^. Volcanic rocks spread throughout the region and continental sediments ranging in age from Late Miocene to the present form the units of last geological period^1,26,28,29,42,44,47^.

The Eastern Anatolian plateau, one of the youngest and widest plateaus of the world, represents a suture zone at which the northern and southern branches of Neotethys come together^9,10^. The basement of the Eastern Anatolian plateau consists of micro-continents stacked together between Late Cretaceous–Early Tertiary and separated from each other by ophiolite belts and accretion complexes^1,62^. Five different tectonic blocks are recognized in Eastern Anatolia: the Eastern Rhodope-Pontide fragment, the Northwest Iranian fragment, the Bitlis-Pötürge unit, Autochthonous units of the Arabian continent and the Eastern Anatolian Accretionary Complex. Except for the Eastern Anatolian Accumulation Complex, all other tectonic blocks correspond to the microcontinents mentioned above^29^. The Eastern Anatolian Accretionary Complex (EAAC) represents the remnant of a huge subduction accretion complex located between the Rhodop–Pontide and the Bitlis–Pötürge micro-continent, formed within Late Cretaceous–Oligocene^26^.

**Figure 2:**
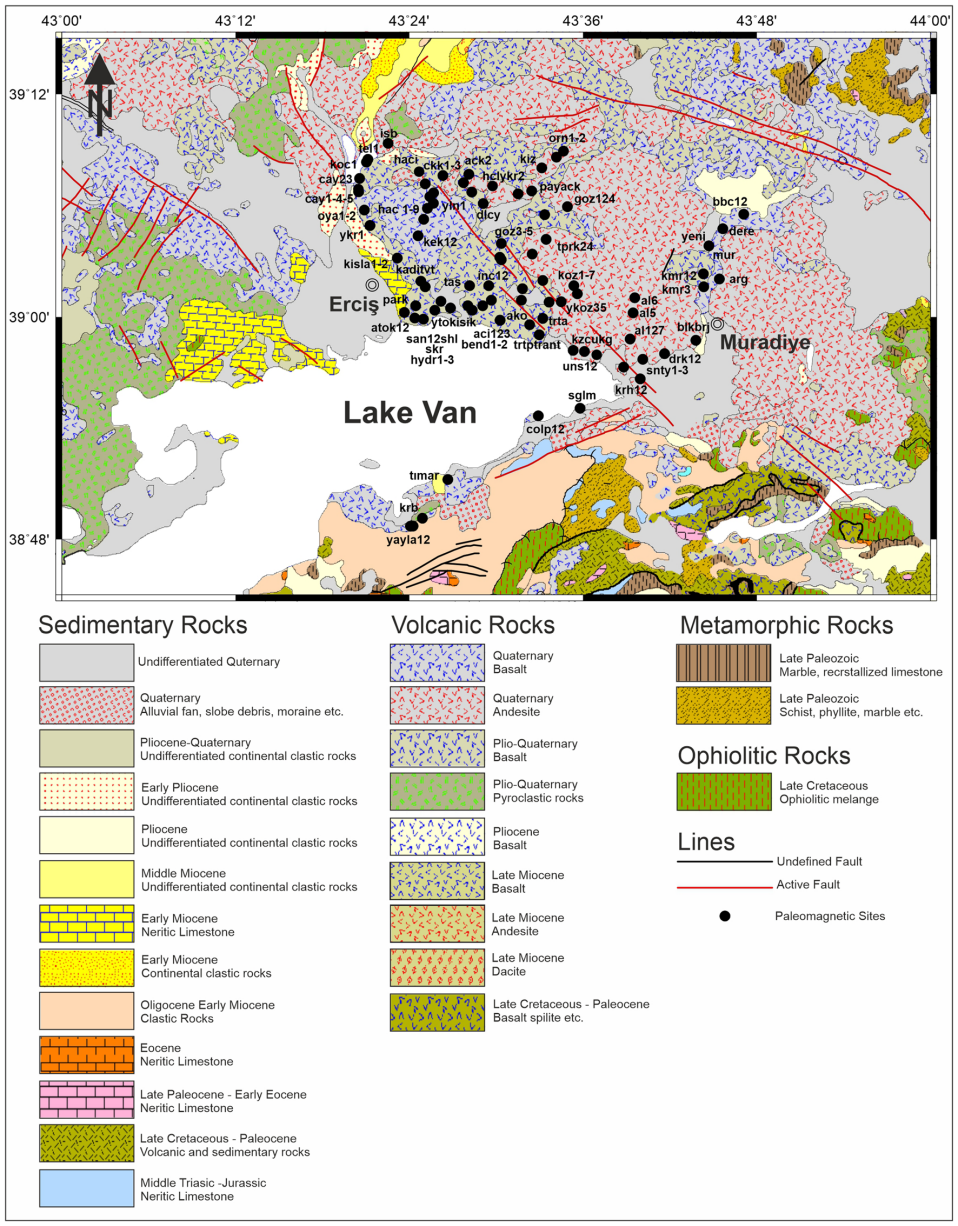
Geological map of the study area including paleomagnetic sample sites of our study. Map were re-arranged from^85^, 1/500.000 geological map, by using the CorelDRAW Graphics Suite (Education License), Version 2021, (https://www.coreldraw.com/en/).

The collision between the Eurasian and Arabian continents occurred in Serravallian (~ 13–11 Ma)^9,10,29^. Volcanic activity started right after the rapid block uplift of Eastern Anatolia and produced different volcanic products that spread throughout the region (Fig. 2)^27,29,47,63,64^. Volcanic activity first started around Erzurum-Kars plateau with calc-alkaline lavas in the north, then migrated to the south-southeast and became more alkaline^28,29^. This volcanic activity has produced a large volume of volcanic material that covers almost two-thirds of the area and in some places exceeds 1 km in thickness. Besides fissure eruptions in volcanic activity, there are also many volcanic centers (e.g. Ağrı, Nemrut and Tendürek Mountains).

The first stage of volcanism in Middle Miocene produced Aladağ Volcanics, which are widespread to the north-northeast of Lake Van^42,65,66^. The next stage of volcanism started at the beginning of Late Miocene (~ 10 Ma). Located in the area between the Ilıca and Deliçay rivers, these rocks have a widely varying chemical composition from basalts and sparsely trachybasalts to dacites^42^. Pliocene volcanism started 5.8–3.7 Ma as trachytic ignimbrites and tuffs that cover a vast area northwest of the town of Erciş. The activity of the Etrüsk Volcano up to trachydacites, trachytes and trachyandesites is the last phase of Pliocene magmatic activity. The activity of this volcano covers a time period between 4.3 and 3.7 Ma^42–44^. Quaternary volcanism in the north of Lake Van, including Girekol Volcano and Karnıyarık cinder cone, started ~ 1 Ma and this young volcanism ended ~ 400 ka.

We collected paleomagnetic oriented samples between 2015 and 2017 from the volcanic rocks whose ages and geochemical compositions have already been known from radiometric aging and petrography methods^42,43^ to determine the tectonic evolution of the North of Lake Van. The orientations of the samples were determined using magnetic/sun compasses and samples were drilled using a portable oil-powered motorized drill with water-cooling, diamond-coated, non-magnetic drill bits. The paleomagnetic samples were collected around the northeast of Lake Van from Pleistocene volcanics (38 sites), Pliocene volcanics (82 sites), and Late Miocene volcanics (14 sites). To increase the number of samples at paleomagnetic sites and make them statistically more reliable, we have combined two or more very close sites from the same age and rock type to create sites with a larger sample count. Thus, we have 32 Pleistocene, 58 Pliocene and 10 Late Miocene sites (Fig. 2).

## Laboratory studies

The paleomagnetic laboratory studies were performed at the “KANTEK Paleomagnetism Laboratory” which is a collaborative laboratory of Boğaziçi University and Istanbul Technical University (ITU). Cylindrical core samples were cut into standard paleomagnetic specimens (2246 in total) and were subjected to stepwise thermal demagnetization using “Magnetic Measurements MMTD-60” thermal demagnetizer. The thermal demagnetization is applied in increments of 25–50 °C up to a maximum temperature of 650 °C. Molspin spinner magnetometer was used to measure magnetization directions and intensities of the Natural Remanent Magnetization (NRM) after each step of thermal demagnetization. Principal component analysis^67^ and orthogonal vector diagrams^68^ were used to describe the Characteristic Remanent Magnetization (ChRM). The ChRM directions and their statistical parameters for each site were determined using standard Fisher statistical analysis with 45° cut-off^69^. Errors in declination (ΔDx) and inclination (ΔIx) were calculated from the A95 of the Virtual Geomagnetic Pole (VGP) distribution for all sites. The criteria defined by Deenen et al.^70,71^ indicate that the determined A95 value of the VGP distribution needs to be between the N-dependent values of A95min and A95max to represent the paleosecular variations (PSV) in the geomagnetic field. Remasoft 3.0 Paleomagnetic Data Browser and Analyzer software have been used to interpret the demagnetization diagrams. The reversal test and its classification developed by McFadden and McElhinny^72^ were used to determine whether the two distributions with the positive and negative polarity means have a common mean direction.

Rock magnetic studies (Isothermal remanent magnetization-IRM and high-temperature susceptibility-HTS) have been carried out to determine the magnetic minerals responsible for permanent magnetization and to determine the change with the coercive force in addition to paleomagnetism studies. All measurements of rock magnetic studies were done in Yılmaz İspir Paleomagnetism Laboratory at Istanbul University-Cerrahpaşa, Department of Geophysics.

HTS measurements have been carried out on 16 representative samples by heating in room conditions. The heating and cooling phases of the grinded sample between room temperature (24 °C) and 650 °C were done using the Bartington MS2 Susceptibility/Temperature System with Bartington MS2 susceptibility meter. IRM studies were performed on pilot samples from each rock type to detect the minerals responsible for the magnetization in the rock.

## Analysis results

### Rock magnetism studies

**Figure 3:**
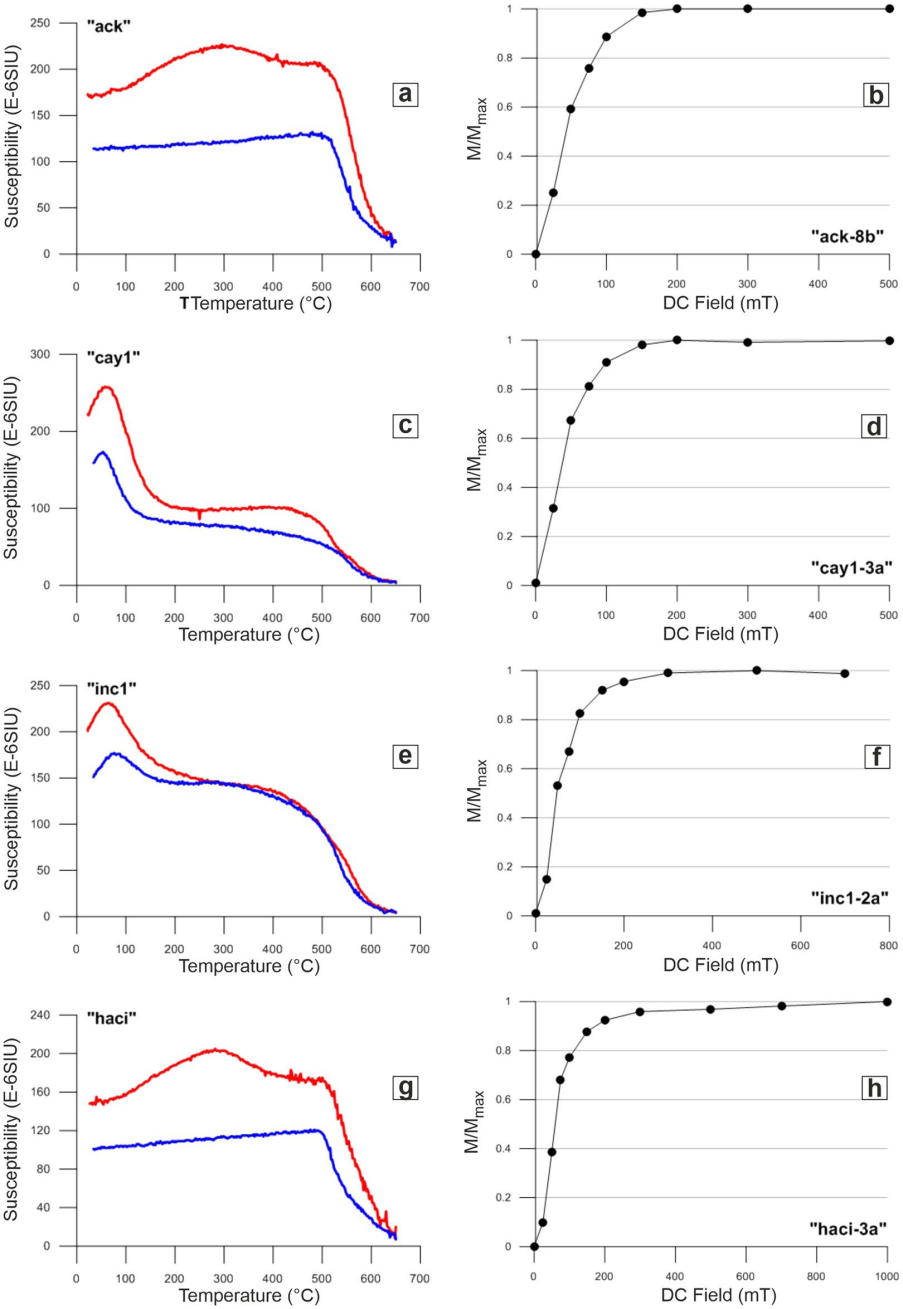
High-temperature susceptibility curves (**a**,**c**,**e**,**g**) and normalized IRM acquisition curves (**b**,**d**,**f**,**h**) for representative samples. (normalized IRM acquisition curves were obtained from one sample of the site for which HTS was measured. e.g. haci3–haci-3a).

We made at least one HTS measurement for each rock type to determine the rocks’ magnetic properties and to identify its magnetic behavior at different temperatures. The analysis of HTS measurements performed in 16 samples of different age and rock types show three different types of behavior. The red curves given in Fig. 3 illustrate the heating phase and the blue curves indicate the cooling phase.

Low Curie temperatures are observed between 150 and 250 °C in one of the sample groups (cay1, colp, san2, koz5, inc1), which is an indication of presence of titanomagnetite component in these samples.

Some of the samples show reversible behavior (atok, blk, cay1, inc1, koz5, ykoz5, ykr) with no remarkable difference between heating and cooling curves. On the other hand, the heating and cooling curves of some samples differ considerably, in relation to remarkable degree of alteration (ack, kad2, cay4, colp, yayla2). Heating curves depict a reduction of around 400 °C in two samples (ack, haci), which indicates the transformation to maghemite or the existence of Ti-rich titanomagnetite.

**Figure 4:**
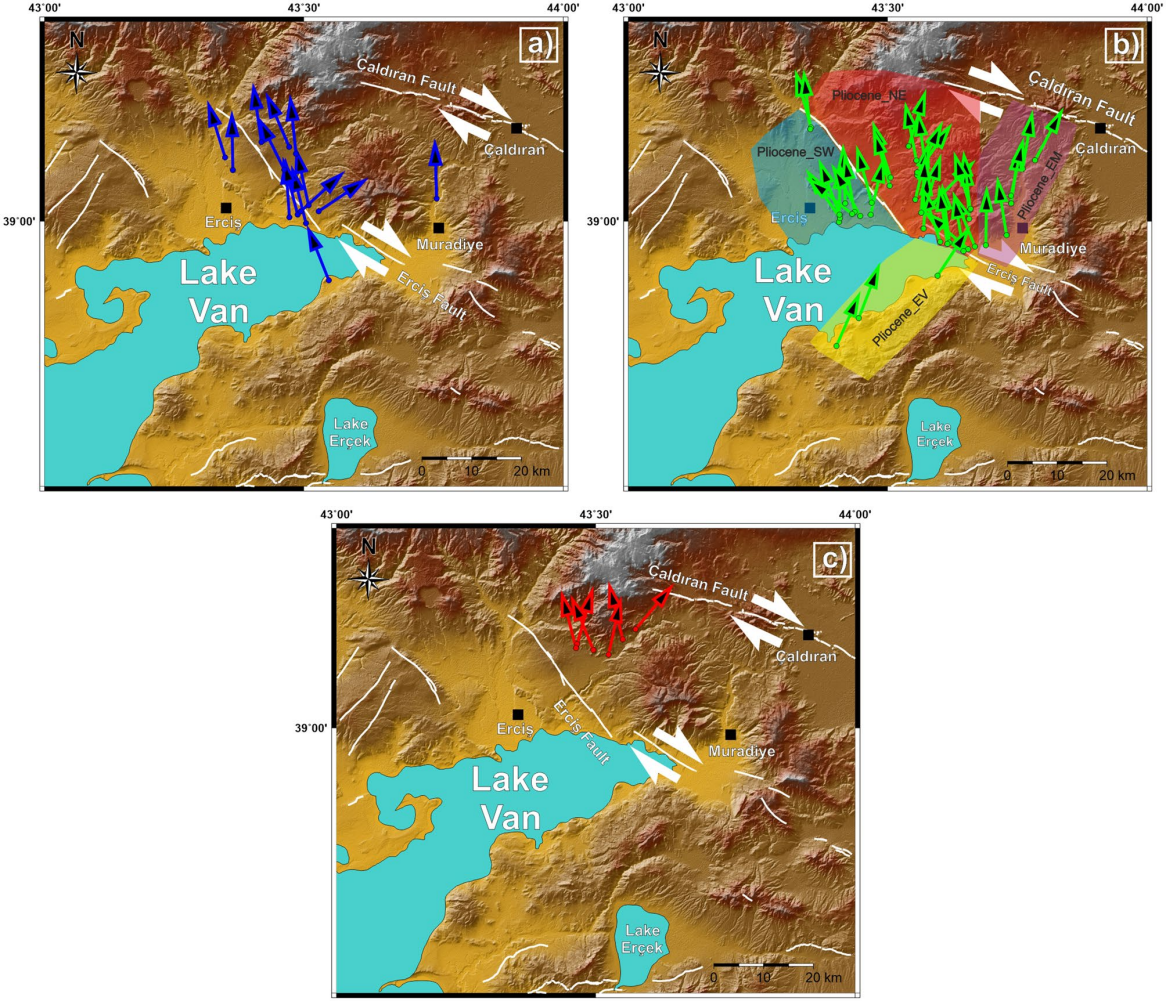
(**a**) Mean remanent magnetization directions of Pleistocene volcanic rocks. (**b**) Mean remanent magnetization directions of Pliocene volcanic rocks**. (c**) Mean remanent magnetization directions of Late Miocene volcanic rocks. Maps were created by using the Generic Mapping Tools software, version 5.1.1. (https://www.soest.hawaii.edu/gmt/)^84^.

IRM measurements were applied to the samples taken from 33 sites representing each of the volcanic rocks located to the north of Lake Van. IRM curves in Fig. 3 indicate that magnetite is the responsible mineral for magnetization. Two different types of behavior are observed in the IRM acquisition curves. The first type of IRM curve is characterized by low to moderate coercivity phases, reaching saturation between 0.1 and 0.3 T. There are 21 sites (Arg, Bend, Colp, Inc1, Orn, etc.) where the saturation is observed in IRM curves and magnetite mineral is dominantly responsible for their magnetization. The second type of IRM curve is characterized by a rapid increase of magnetization in low fields at first (up to 1 T) and then a small increase without complete saturation at 1 T (Fig. 4). There are 12 sites (Ykoz, Ykr, Haci, Skr, Tprk4, etc.) whose IRM curves are classified as the second type, indicating that both magnetite and hematite are responsible for the magnetization. Hematite was not dominant in any of the samples.

### Paleomagnetic studies

In the study area, there are volcanics of different rock types in the Late Miocene–Pleistocene range. During the interpretation of the paleomagnetic data, we divided these volcanics into 3 different age groups as Late Miocene, Pliocene and Pleistocene. Late Miocene volcanism in the region is represented by products from Aladağlar and Meydan Mountains. These volcanics are located to the north of the Zilan Valley and Etrüsk Volcano within the vicinity of the Tendürek Mountain (N-NW of Erciş Village). Late Miocene-aged sites are generally located in the north-northeast of Erciş district. The Pliocene-aged volcanics, which are the most common rocks in the study area, are around Van, Erciş, Etrüsk Mountain and Muradiye. Pleistocene volcanics are located in the north of the Erciş district and the west of Etrüsk Mountain as Girekol, Yüksektepe and Karnıyarık volcanics. Pleistocene aged sites are also located in these regions.

#### Group mean directions

Pleistocene aged rocks in the north of Lake Van have been sampled from 32 paleomagnetic sites. Except for 11 statistically unreliable paleomagnetic sites which are excluded from evaluation, all sites have normal polarities (Table 1). Seven sites show suspicious directions that are diverging from the average distribution and sites with A95 values outside the envelope of A95min and A95max were not taken into evaluation. Eight of the 14 remaining sites with reliable magnetization were in paleo-horizon and no tilt correction was applied. The remaining six sites are tilt corrected and the results of all 14 sites are given in Table 1.

**Table 1:** Paleomagnetic results from Late miocene to pleistocene volcanic rocks.

Site	Lat. (°)	Lon. (°)	Age	D_n_/T_n_	D_g_ (°)	I_g_ (°)	D_s_ (°)	I_s_ (°)	R	k	α_95_ (°)	A95	A95min	A95max	Unit
arg	39,034	43,756	Pleistocene	8/8	0.6	43.1	**358.2**	**48.5**	7.91	78.77	6.3	7.2	5.5	24.1	Tephrite
oya2	39,104	43,342	Pleistocene	No mean direction could be obtained											Hawaiit
yln	39,112	43,472	Pleistocene	7/8	**336.9**	**47.4**			6.78	26.73	11.9	12.6	5.5	24.1	Hawaiit
cay1*	39,112	43,341	Pleistocene	7/11	326.8	6.1	**326.1**	**12.8**	6.88	50.19	8.6				Hawaiit
ckk*	39,119	43,454	Pleistocene	6/8	282.9	33.1	**286.9**	**32.7**	5.79	23.73	14				Hawaiit
hac1*	39,121	43,428	Pleistocene	6/9	**288.3**	**26.1**			5.8	25.48	13.5				Hawaiit
hac2*	39,12	43,424	Pleistocene	6/8	**191.8**	**44.2**			5.77	21.61	14.7				Hawaiit
hac3	39,119	43,418	Pleistocene	8/9	354.2	41.4	**351.8**	**49.1**	7.9	73.46	6.5	7.8	5.2	22.1	Hawaiit
hac4	39,112	43,425	Pleistocene	No mean direction could be obtained											Hawaiit
hac5	39,107	43,423	Pleistocene	No mean direction could be obtained											Hawaiit
hac7	39,102	43,416	Pleistocene	No mean direction could be obtained											Hawaiit
bend	39,006	43,472	Pleistocene	9/9	**356.1**	**55.7**			8.92	101.91	5.1	6.2	5	20.5	Basalt
aci123	38,997	43,504	Pleistocene	19/25	**345.9**	**49.3**			18.17	21.62	7.4	7.4	3.8	13.3	Basalt
koz5	39,015	43,528	Pleistocene	7/8	52	20.6	**55.9**	**25.0**	6.91	63.3	7.6	7.9	5.5	24.1	Hawaiit
inc12	39,01	43,488	Pleistocene	12/16	**345.4**	**50.6**			11.76	46.70	6.4	7.3	4.4	17.1	Basalt
inc4	39.015	43.494	Pleistocene	7/8	**47.5**	**38.1**			6.9	61.28	7.8	7.4	5.5	24.1	Basalt
dlcy	39.102	43.485	Pleistocene	7/10	**354.3**	**55.4**			6.88	48.71	8.7	11.1	5.5	24.1	Basalt
colp12	38.911	43.548	Pleistocene	13/17	**338.4**	**12.7**			13.29	18.24	9.6	8.7	4.2	15.6	Basalt
kad2**	39.032	43.413	Pleistocene	6/8	359.6	43.2			5.99	583.13	2.8	2.2	6.3	29.7	Basalt
oya	39.096	43.348	Pleistocene	8/8	356.5	48	**342.8**	**48.4**	7.93	98	5.6	5.4	5.2	22.1	Basalt
kek12	39.073	43.41	Pleistocene	13/17	335.4	41.9	**333.7**	**45.7**	12.60	30.30	7.7	9.1	4.3	16.3	Basalt
kek3	39.074	43.404	Pleistocene	No mean direction could be obtained											Basalt
hac6	39.101	43.422	Pleistocene	No mean direction could be obtained											Basalt
hac8	39.095	43.415	Pleistocene	No mean direction could be obtained											Basalt
hac9	39.09	43.421	Pleistocene	No mean direction could be obtained											Basalt
tprk5	39.024	43.508	Pleistocene	No mean direction could be obtained											Basalt
ykr	39.077	43.363	Pleistocene	7/8	**359**	**51.6**			6.83	35.58	10.3	12.4	5.5	24.1	Basalt
tprk67	39.025	43.53	Pleistocene	17/19	**347.6**	**49.9**			16.51	32.54	6.3	6.4	3.9	13.8	Basalt
koz1	39.025	43.541	Pleistocene	No mean direction could be obtained											Basalt
ckk2**	39.127	43.438	Pleistocene	8/8	**355.9**	**44.2**			7.98	345.31	3	3.5	5.2	22.1	Basalt
koz4*	39.032	43.549	Pleistocene	6/10	20.1	75.7	**295.4**	**57.4**	5.82	28.48	12.8				Basalt
ckk3	39.125	43.432	Pleistocene	No mean direction could be obtained											Basalt
**Pleistocene group mean**				**11**	**348.0**	**50.4**			**10.94**	**166.85**	**3.5**	**2.5**	**1.8**	**4.3**	
bbc12	39.092	43.784	Pliocene	16/19	**208.3**	− **57.5**			15.62	39.00	6.0	6.6	4.0	14.3	Latite
kmr12	39.027	43.738	Pliocene	16/17	**188.1**	− **43.1**			15.69	48.14	5.4	4.5	4.0	14.3	Rhyolite
kmr3mur	39.039	43.738	Pliocene	16/20	180.9	− 20.1	**192.2**	− **35.0**	14.54	30.52	7.0	7.2	4.1	14.9	Rhyolite
drk12	38.964	43.689	Pliocene	17/17	**180.9**	− **53.0**			16.81	82.57	3.9	4.6	3.9	13.8	Rhyolite
dere	39.079	43.76	Pliocene	8/9	**9.7**	**32.6**			7.89	66.32	6.9	6.5	5.2	22.1	Latite
blkbrj	38.979	43.729	Pliocene	14/16	**171.0**	− **53.3**			13.74	49.66	5.7	7.2	4.2	15.6	Rhyolite
**Pliocene_EM group mean**				**6**	**188.3**	− **46.2**			**5.87**	**38.84**	**10.9**	**3.4**	**2.2**	**5.6**	
al5	39.006	43.656	Pliocene	No mean direction could be obtained											Trachyte
koz7**	39.013	43.56	Pliocene	8/8	181.4	− 48.3	**198.9**	− **53.5**	7.99	818.97	1.9	2.5	5.2	22.1	Latite
al127	39.004	43.657	Pliocene	20/21	**176.9**	− **52.7**			19.08	20.56	7.4	8.9	3.6	12.4	Latite
al3	39.017	43.659	Pliocene	12/14	**173.6**	− **51.4**			104.51	104.51	4.3	5.2	4.4	17.1	Trakidacite
al4	39.019	43.66	Pliocene	7/8	169.2	− 48.5	**163.8**	− **52.5**	6.92	75.22	7	7.3	5.5	24.1	Trachy-andesite
al6*	38.978	43.651	Pliocene	7/8	**99.5**	− **55.2**			6.9	60.73	7.8				Latite
goz124	39.092	43.555	Pliocene	22/26	**169.0**	− **50.8**			21.25	28.08	6.0	7.1	3.6	12	Latite
goz3	39.066	43.505	Pliocene	9/9	171.6	− 46.5	**162.3**	− **48.7**	8.91	88.8	5.5	6.3	5.0	20.5	Trachyte
koz6	39.013	43.564	Pliocene	7/8	**184.3**	− **52.4**			6.96	140.47	5.1	6.3	5.5	24.1	Trachyte
koz2	39.033	43.553	Pliocene	6/8	**13.1**	**41**			5.77	22.2	14.5	15	5.9	26.5	Trachyte
goz5	39.053	43.504	Pliocene	6/7	**344.4**	**50.5**			5.9	48.54	9.7	10.5	5.9	26.5	Latite
pay2	39.113	43.54	Pliocene	6/6	**16.9**	**52.5**			5.8	24.64	13.8	14.7	5.9	26.5	Basalt
snty2	38.958	43.655	Pliocene	8/8	**131.2**	− **70.7**			7.96	196.3	4.0	5.9	5.2	22.1	Trachyte
snty3*	38.967	43.659	Pliocene	7/8	335.8	− 67.1	**333.2**	− **65.5**	6.83	34.78	10.4				Latite
tprk1	39.079	43.573	Pliocene	13/17	**172.0**	− **60.8**			13.65	37.38	6.6	8.0	4.3	16.3	Latite
tprk24	39.074	43.557	Pliocene	17/18	225.1	− 38.9	**215.5**	− **61.3**	16.83	92.68	3.7	5.5	3.9	13.8	Trakidacite
tprk3	39.07	43.557	Pliocene	7/8	**205.2**	− **51.8**			6.8	30.59	11.1	14.3	5.5	24.1	Trakidacite
ykoz2	39.028	43.589	Pliocene	8/9	198.2	− 39.8	**224.1**	− **69.8**	7.91	78.03	6.3	9.5	5.2	22.1	Latite
ykoz4	39.014	43.574	Pliocene	8/8	177.5	− 40	**168.9**	− **51.3**	7.9	71.24	6.6				Trachyte
ykoz35	39.015	43.571	Pliocene	13/15	**196.2**	− **55.1**			12.71	41.81	6.5	8.9	4.3	16.3	Trachyte
yeni*	39.071	43.756	Pliocene	8/8	**10.2**	− **62.3**			7.61	18.14	13.4				Latite
**Pliocene_NE group mean**				**15**	**181.8**	− **56.5**			**14.57**	**32.82**	**6.8**	**2.7**	**1.5**	**3.1**	
krh12	38.962	43.668	Pliocene	16/16	**158.1**	− **68.1**			15.79	70.84	4.4	7.2	4.0	14.3	Rhyolite
kzcukg	38.968	43.619	Pliyosen	15/15	**172.9**	− **61.4**			14.81	75.61	4.4	5.9	4.1	14.9	Trachyte
isb*	39.161	43.371	Pliocene	6/7	**96.2**	**69.7**			5.74	19.14	15.7				Trachyte
ako**	38.993	43.538	Pliocene	7/7	**195.5**	− **66.8**			6.99	455.9	2.8	4.5	5.5	24.1	Trachyte
inc3*	39.028	43.491	Pliocene	6/8	**129.4**	− **40.4**			5.89	45.72	10				Trachy-andesite
kisla*	39.056	43.383	Pliocene	5/8	**248.5**	− **2.1**			4.88	34.57	13.2				Trachyte
kisla2**	39.053	43.386	Pliocene	7/8	**182.5**	− **28.7**			5.987	380.4	3.4	2.8	5.9	26.5	Trachyte
cay5	39.13	43.354	Pliocene	No mean direction could be obtained											Hawaiit
trta	38.988	43.57	Pliocene	No mean direction could be obtained											Trachyte
koz3	39.036	43.554	Pliocene	No mean direction could be obtained											Rhyolite
kzc2	38.966	43.615	Pliocene	7/7	167	− 70.2	**171.8**	− **56.1**	6.96	170.20	4.6	5.5	5.5	24.1	Trachyte
trtptrant	38.989	43.569	Pliocene	22/24	**184.2**	− **58**			21.46	39.24	5.0	6.6	3.5	11.7	Trachyte
uns12	38.969	43.601	Pliocene	15/16	**162.0**	− **64.1**			14.91	157.4	3.1	4.2	4.1	14.9	Trachyte
tas	39.028	43.469	Pliocene	8/8	**165.1**	− **58.7**			7.96	174.2	4.2	5.5	5.2	22.1	Latite
park	39.012	43.398	Pliocene	No mean direction could be obtained											Trachyte
snty	38.955	43.646	Pliocene	8/8	**170.6**	− **70.3**			7.964	193.1	4.0	5.8	5.2	22.1	Trachyte
hydr12yko	39.006	43.421	Pliocene	10/10	**169.7**	− **55.2**			23.7	18.47	6.9	7.7	3.4	11.4	Trachyte
atok12	38.999	43.406	Pliocene	13/17	**1.2**	**66.5**			12.88	96.95	4.2	6.2	4.3	16.3	Trachyte
san12shl	39.005	43.408	Pliocene	24/24	**145.1**	− **62.2**			23.69	73.73	3.5	4.8	3.4	11.1	Trachyte
ytokisik	39.01	43.407	Pliocene	15/15	**159.6**	− **54.4**			14.85	95.44	3.9	5.1	4.1	14.9	Trachyte
bend2	39.01	43.467	Pliocene	8/8	**192.9**	− **57.7**			7.95	131.33	4.9	6	5.2	22.1	Latite
skr	39.008	43.447	Pliocene	8/8	**151.1**	− **55.5**			7.91	74.55	6.5	6.3	5.2	22.1	Trachyte
hydr3	39.011	43.431	Pliocene	8/8	149.6	− 77.3	**128.5**	− **68.2**	7.84	44.3	8.4	13.2	5.2	22.1	Trachyte
kaditvt	39.027	43.418	Pliocene	13/13	**177.9**	− **54.9**			12.91	130.9	3.6	4.4	4.3	16.3	Trachyte
tel	39.141	43.352	Pliocene	8/10	177.8	− 64.9	**164.6**	− **53.2**	7.95	151.54	4.5	5.3	5.2	22.1	Hawaiit
koc	39.139	43.351	Pliocene	6/8	**173.6**	− **45.2**			5.9	52.38	9.3	8.7	5.9	26.5	TrakiBasalt
**Pliocene_SW group mean**				**17**	**167.0**	− **61.0**			**16.75**	**65.30**	**4.4**	**2.3**	**1.5**	**3.0**	
sglm	38.918	43.596	Pliocene	5/8	**33.4**	**64.5**			4.77	17.15	19	26.2	6.3	29.7	Trachyte
yayla12	38.812	43.401	Pliocene	14/15	**23.6**	**71.8**			13.8	65.71	4.9	8.6	4.2	15.6	Basalt
krb*	38.865	43.465	Pliocene	7/8	**130.5**	**64**			6.98	243.61	3.9				Basalt
nors**	38.819	43.415	Pliocene	9/9	**44.8**	**53.8**			8.98	437.73	2.5	3	5	20.5	Basalt
timar	38.854	43.444	Pliocene	7/8	18.3	54.3	**20.9**	**59**	6.79	28.52	11.5	7.3	5.9	26.5	Basalt
**Pliocene_EV group mean**				**3**	**25.8**	**65.2**			**2.98**	**133.13**	**10.7**	**6.2**	**2.9**	**9**	
cay23**	39.115	43.341	L.Miocene	16/17	**189.8**	− **54.0**			15.96	342.60	2.0	2.5	4.0	14.3	Ignimbrite
cay4**	39.13	43.35	L.Miocene	8/8	**192.1**	− **57**			7.99	713.73	2.1	2.8	5.2	22.1	Trachyte
haci**	39.13	43.411	L.Miocene	8/8	2.2	48.9	**338.8**	**55.7**	7.99	472.18	2.6	3	5.2	22.1	Trachy-andesiticTuff
hclykr2	39.117	43.495	L.Miocene	17/17	166.1	− 29.2	**157.0**	− **50.0**	16.85	111.90	3.4	4.0	3.9	13.8	Dacite
payack	39.11	43.525	L.Miocene	16/16	**192.4**	− **57.0**			15.71	51.09	5.2	7.4	4.0	14.3	Dacite
ack2	39.126	43.464	L.Miocene	7/8	**195.6**	− **53.6**			6.96	133.74	5.2	6.5	5.5	24.1	Hawaiit
orn	39.149	43.577	L.Miocene	7/8	36	38.8	**39.9**	**61.6**	6.95	115.05	5.7	7.7	5.5	24.1	Andesite
orn2*	39.152	43.583	L.Miocene	7/8	80	− 26	**110.8**	− **45.6**	6.88	51.71	8.5				Basalt
yln2kiz2	39.12	43.462	L.Miocene	11/14	**165.4**	− **40.4**			10.47	18.92	10.8	12.7	4.6	18.1	Basalt
kiz	39.133	43.552	L.Miocene	7/7	**166.2**	− **49.4**			6.92	79.02	6.8	6.7	5.5	24.1	Andesite
**LateMiocene group mean**				**6**	**179.7**	− **53.9**			**5.82**	**27.06**	**13.1**	**3.5**	**1.9**	**4.6**	

The site mean directions are highlighted in bold.

*Lat* latitude of the sites; *Lon* longitude of the sites; *D*_*n*_*/T*_*n*_* T*_*n*_ denotes the number of samples per locality, *D*_*n*_ the number of samples used for site mean calculation, *D*_*g(s)*_*, I*_*g(s)*_ Declination and Inclination angle in geographic (before tilt correction) and stratigraphic (after tilt correction) coordinates, respectively, *R* resultant vector, *k* precision parameter, *α*_*95*_ 95% confidence circle. *A95* cone of confidence determined from the mean VGP direction, *A95min/A95max* minimum/maximum value of the A95. The age and the lithology information of all paleomagnetic sites are from^42^ and^43^. The sites with the following characteristics were excluded from further analyses:

*Sites showing suspicious directions divergent from the average distribution.

**Sites with A95 values outside the envelope of A95min and A95max.

These 14 normal polarity sites in Pleistocene volcanics show a mean direction in geographic coordinates of D = 350.0° and I = 48.9° with statistical parameters k = 113.76 and α_95_ = 4.3° and in the stratigraphic coordinates D = 348.0° and I = 50.4° with statistical parameters N = 14, k = 166.85 and α_95_ = 3.5° (Table 1; Fig. 4a).

A total of 58 paleomagnetic sites have been sampled from Pliocene aged rocks in the north of Lake Van. Five sites are excluded from evaluation because of statistical unreliability (Table 1). A total of 11 sites showing suspicious directions deviating from the average distribution and sites with A95 values outside the envelope of A95min and A95max were not taken into evaluation.

Nine of the Pliocene-aged sites have normal polarity and the rest have reverse polarity. A positive reversal test is obtained with classification of “B”^72^ and the k ratio is 1.3. The normal polarity sites (N = 9) give a clockwise rotation (D = 11.7°, I = 55.8°, R = 8.7 and k = 30.4). Reverse polarity sites (N = 33) give a counterclockwise rotation (D = 178.4°, I = − 58.2°, R = 32.1 and k = 36.4). The observed angular difference (γ = 7.6°) is smaller than the critical angular difference (γ_c_ = 9.2°) indicating that the secondary overprints have been removed in the Pliocene paleomagnetic data.

Pliocene volcanic rocks are geographically close to each other and split into four different groups during evaluation: The south of the Erciş Fault (Pliocene_SW), the north of the Erciş Fault (Pliocene_NE), around Muradiye (Pliocene_EM), and the north of the Van (Pliocene_EV) (Table 1; Fig. 4b).

For the Pliocene sites in the East of Van (Pliocene_EV), mean directions of the group are found to be D = 24.4° and I = 63.7° with statistical parameters N = 3, k = 73.19 and α_95_ = 14.5°, which are updated as D = 25.8° and I = 65.2° with statistical parameters N = 3, k = 133.13 and α_95_ = 10.7° after tilt correction (Table 1; Fig. 4b). All of three sites have normal polarity. For the Pliocene sites in the South of the Erciş Fault (Pliocene_SW), group mean direction is calculated as D = 349.3° and I = 61.2° with statistical parameters N = 17, k = 70.13 and α_95_ = 4.3° before tilt correction, D = 347° and I = 61.0° with statistical parameters N = 17, k = 65.30 and α_95_ = 4.4° after tilt correction. Two sites have normal and 15 sites have reverse polarity (Table 1; Fig. 4b).

Pliocene sites in the North of the Erciş Fault (Pliocene_NE) show mean direction of the group as D = 3.8° and I = 51.8° with statistical parameters N = 16, k = 29.88 and α_95_ = 6.9°, and D = 0.9° and I = 56.2° with statistical parameters N = 16, k = 34.21 and α_95_ = 6.4° after tilt correction (Table 1; Fig. 4b). Four sites have normal and 12 sites have reverse polarity.

The group near the Muradiye is named Pliocene_EM and has six sites. The mean direction of this group is calculated as D = 5.9° and I = 43.8° with statistical parameters N = 6, k = 38.84 and α_95_ = 10.9° before tilt correction, D = 8.3° and I = 46.2° with statistical parameters N = 6, k = 38.84 and α_95_ = 10.9° after tilt correction. One site has normal and five sites have reverse polarity (Table 1; Fig. 4b).

Late Miocene aged rocks in the north of Lake Van were sampled from 10 paleomagnetic sites. There is not any statistically unreliable paleomagnetic sites (Table 1). A total of four sites showing suspicious directions diverging from the average distribution and sites with A95 values outside the envelope of A95min and A95max were not taken into evaluation.

**Figure 5:**
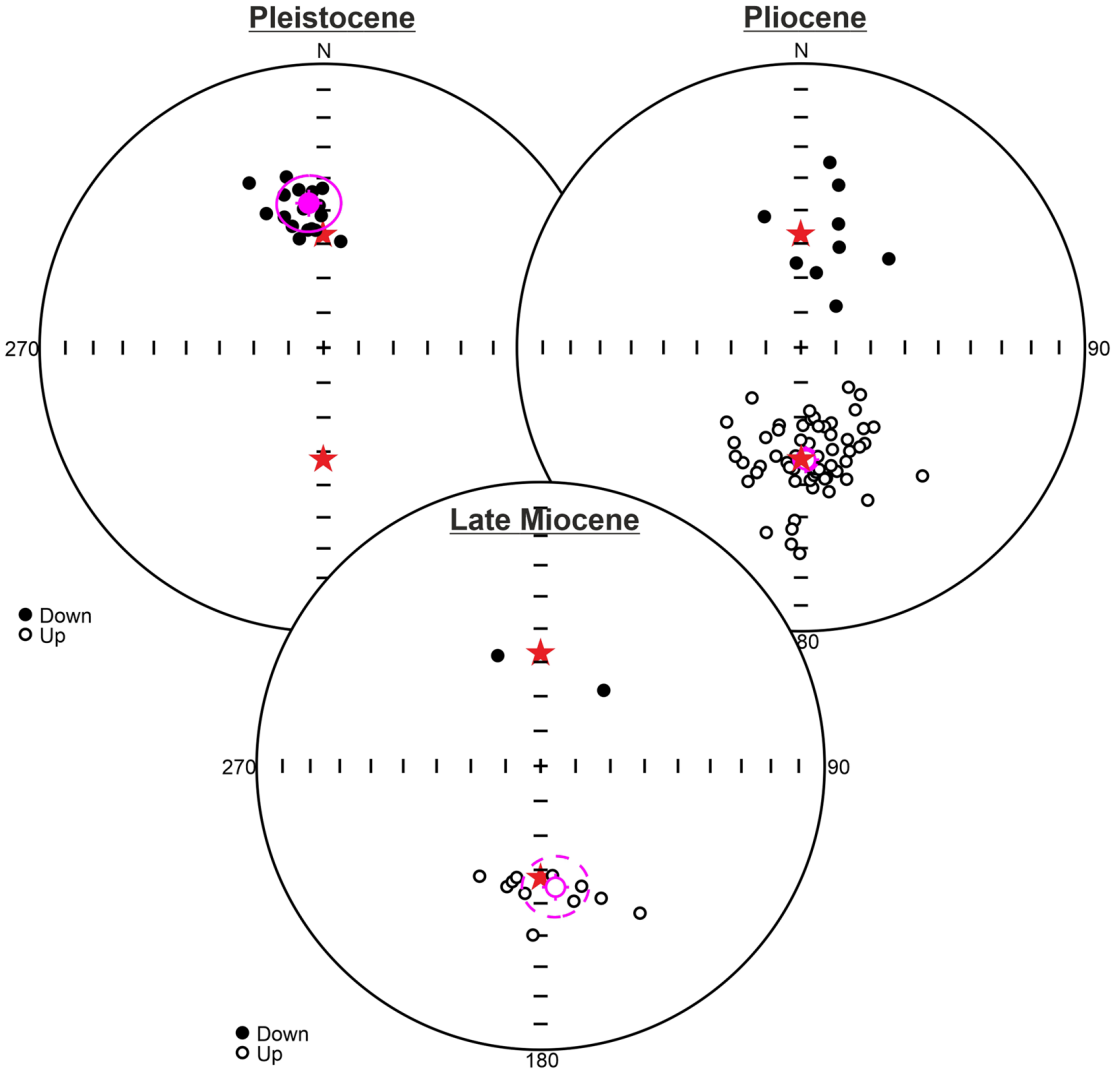
Paleomagnetic mean directions of the north of Lake Van in different time intervals (Pleistocene, Pliocene and Late Miocene). Red stars represent the current magnetic field direction and inclination angle (D/I = 0°/58°) in the latitude where the study area is located.

Late Miocene rocks (N = 6 sites) yield D = 2.6°, I = 46.5° with statistical parameters k = 19.49, α_95_ = 15.6° in in-situ coordinates, and D = 359.7°, I = 53.9° with statistical parameters k = 27.06 and α_95_ = 13.1° after tilt correction from five reverse and one normal polarity sites (Table 1; Fig. 4c). Figure 5 shows stereographic projections and statistical parameters of different aged paleomagnetic groups after tilt corrections.

#### Paleosecular variation

Paleosecular variation (PSV) should be averaged in paleomagnetic studies, so that the paleomagnetic directions show only tectonic movement^70^. PSV should be considered unreliable if A95 values are above or below the A95min and A95max limits^70^. In this study VGPs were calculated from paleomagnetic rotations for each site and group. A95 values for all of the groups and the most of the individual samples fall within the A95min/A95max confidence envelope; samples otherwise were not included within the evaluation. Therefore, it is plausible to consider that PSV is adequately averaged in our paleomagnetic data set.

**Figure 6:**
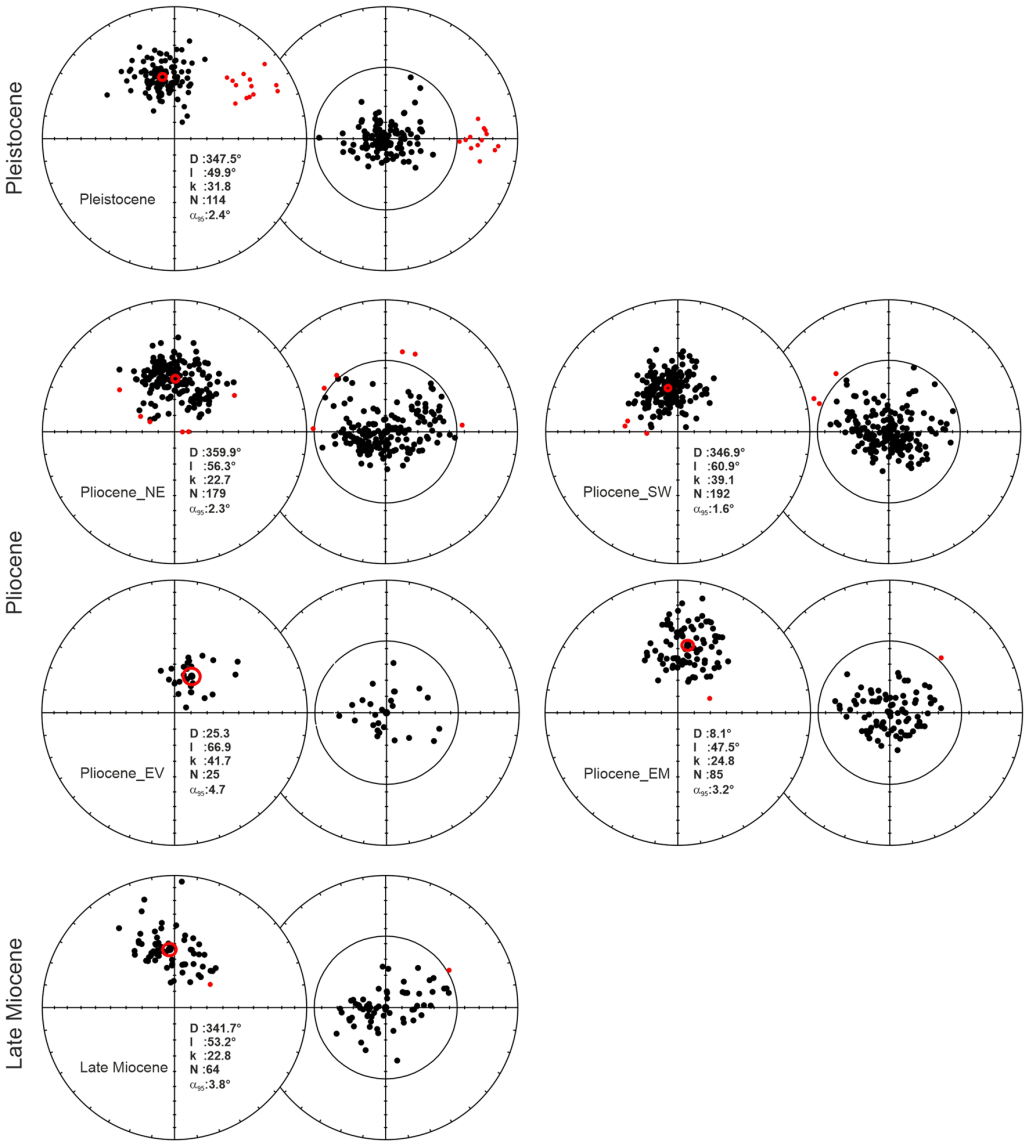
Equal area projections of the ChRM and VGP directions following Deenen’s criteria^70^, respectively. Red circles indicate α95 cone of confidence and solid red dots indicate rejected results. All directions are converted to normal polarity.

A95 values of the whole group mean directions of Late Miocene–Pleistocene aged volcanic rocks are within the required A95min–A95max envelope. Equal area projections of the ChRM and VGP directions for all groups are shown in Fig. 6.

## Discussion

**Figure 7:**
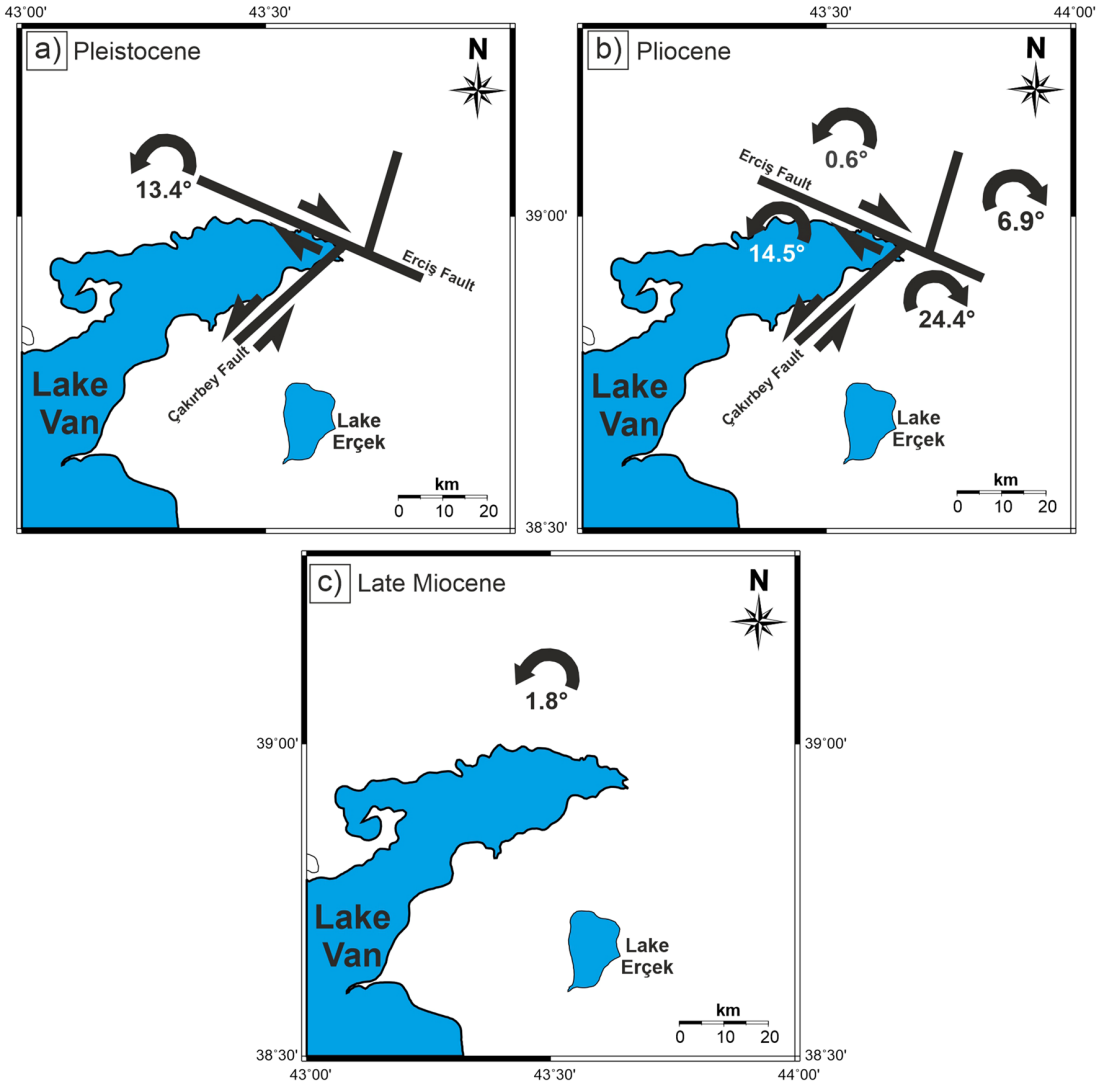
Rotations of blocks in the study area. (**a**) Rotation of the Pleistocene sites, (**b**) Rotations of Pliocene sites and, (**c**) Rotation of Late Miocene sites. Maps were created by using the Generic Mapping Tools software, version 5.1.1. (https://www.soest.hawaii.edu/gmt/)^84^.

The Pleistocene pole position is calculated as 77.4° N, 278.3° E (dp = 4.7, dm = 3.2, α_95_ = 3.5°) showing counterclockwise rotation of R ± ΔR = 13.4° ± 3.8° when compared with the expected stable Eurasia reference pole (λ_ref_, /Φ_ref_ = − 88.5°/353.9°, α_95_ = 1.9°) of^73^ using PMGSC (version 4.2) software^74^ (Fig. 7a). Examining the rotations in the southern part of the Erciş Fault (group Pliocene_SW) implies a counterclockwise rotation: R ± ΔR = 14.5° ± 6.1° (Fig. 7b). On the other hand, no significant rotation (R ± ΔR = 0.6° ± 7.4°) is observed on the northern side of Erciş Fault (group Pliocene_NE) (Fig. 7b). Also, the remarkable clockwise rotation R ± ΔR = 24.4° ± 17.0° to the north of Van (group Pliocene_EV) and clockwise rotation R ± ΔR = 6.9° ± 9.4° of the sites near Muradiye indicate that the prevailing tectonic movement of the region is clockwise (Fig. 7b). In summary, Pliocene paleomagnetic results reveal that there are a lot of remarkable rotation differences around the Erciş Fault, most of which are caused by the Erciş Fault itself. The results from Late Miocene volcanic rocks indicate that there is almost no significant rotation (R ± ΔR = 1.8° ± 13.7°) in the region (Fig. 7c). In Table 2, net rotation amounts of other ages and location groups are given respectively. These values were used in the tectonic interpretation of paleomagnetic data obtained in this study. The difference between the observed poles (λ_obs_, ϕ_obs_) and the reference poles and expected tectonic rotations (R) were computed using the pole-space method of Beck^75^ and the 95% confidence limits (ΔR) were determined after Demarest^76^.

**Table 2:** Group mean ChRM directions (D, I, α_95_) after tectonic correction and expected tectonic rotations (R) with respect to the stable Eurasia reference poles^73^.

Group	Site coord. (λ/φ) (°)	Group mean dir D(°)/I(°)	α_95_(°)	VGP λ_obs_(°)/φ_obs_(°)	α_95_(°)	R + ΔR
Pleistocene	39.05/43.54	348.0/50.4	2.5	77.4/278.3	3.9	− 13.4° ± 3.8°
Pliocene South of the Erciş Fault	39.05/43.54	167.0/− 61.0	4.4	79.7/334.5	5.9	− 14.5° ± 6.1°
Pliocene North of the Erciş Fault	39.05/43.54	181.8/− 56.5	6.8	87.6/206.6	7.8	0.6° ± 7.4°
Pliocene near Muradiye	39.05/43.54	188.3/− 46.2	10.9	76.5/190.4	11.2	6.9° ± 9.4°
Pliocene North of the Van	39.05/43.54	25.8/65.2	10.7	69.6/101.7	15.6	24.4° ± 17.0°
Late Miocene	39.05/43.54	179.7/− 53.9	13.1	85.3/226.7	15.3	1.8° ± 13.7°

The reference pole (λref /Φref = 88.5°/ 173.9°, α_95_ = 1.9°) for the stable Eurasia is obtained after^73^. Here α_95_ is the statistical parameters after^69^. R is the angle of vertical axis rotation (positive indicates clockwise rotation) with respect to the direction computed from the stable Eurasia paleomagnetic pole with 95% confidence limit ΔR (after^75^).

Hisarlı et al.^16^ named the VB as a region that includes the Lake Van and its surroundings, from the Karlıova Triple Junction to the east. According to the paleomagnetic data obtained from this study, VB must have been rotating clockwise starting from Late Miocene.

Our study area does not coincide with the area of the other relevant studies^16,45,46^ and covers a relatively narrower area compared to them, allowing the investigation of smaller “micro-block” movements instead of mono-block movements. Our study also includes more paleomagnetic samples and distribution in the north of Lake Van and results show both clockwise and counterclockwise rotations in the area which can be interpreted that the Van Block is divided into micro-blocks that have different rotation directions.

If the lineament (i.e. fault) between the blocks of same aged rocks determined by the paleomagnetic rotations coincide with the lineament observed in the active fault map, it can be said that the slips along the faults by the current earthquakes are the continuations of the past tectonic movements.

According to the paleomagnetic rotations of the Pleistocene volcanic rocks, it can be concluded that the whole region rotated counterclockwise (~ 13.4° ± 3.8°) and moved as a mono-block. Selçuk et al.^77^ claimed that the slip rate of the Çaldıran Fault was ~ 3 mm/year (for 290,000 years) and that the maximum slip was ~ 900 m. The rotation resulting from such a small slip cannot be measured with paleomagnetic data. For this reason, it is not relevant to expect a clockwise rotation in the area between the Çaldıran Fault and the Erciş Fault, two of which are dextral strike-slip faults. Therefore, it is clear from the obtained data that the region has rotated counterclockwise (~ 13.4° ± 3.8°) since Pleistocene.

Rotations from Pliocene rocks are given in Fig. 7.a. To determine the rotations in the Pliocene–Pleistocene time interval, counterclockwise rotation of the Pleistocene rocks (~ 13.4° ± 3.8°) is needed to be rotated clockwise. After this period, Pliocene_NE rocks were rotated ~ 13° clockwise and Late Miocene rocks were rotated ~ 11° clockwise in the Late Miocene–Pleistocene period. It is observed that the Çakırbey Fault is formed before the Erciş Fault (Pliocene) and causes a rotation of ~ 37.8° towards the east of the Erciş Fault. This suggests that the region was subjected to an active rotation in the counterclockwise direction after Late Miocene until the Quaternary.

Emre et al.^78,79^ does not mark an active fault in the northwest of Lake Erçek. However, according to our results, we suggest that the Çakırbey Fault, which is roughly oriented northeast-southwest towards Erciş Fault, must be active (Fig. 7) and thus different rotations may have developed on both sides of this fault.

**Figure 8:**
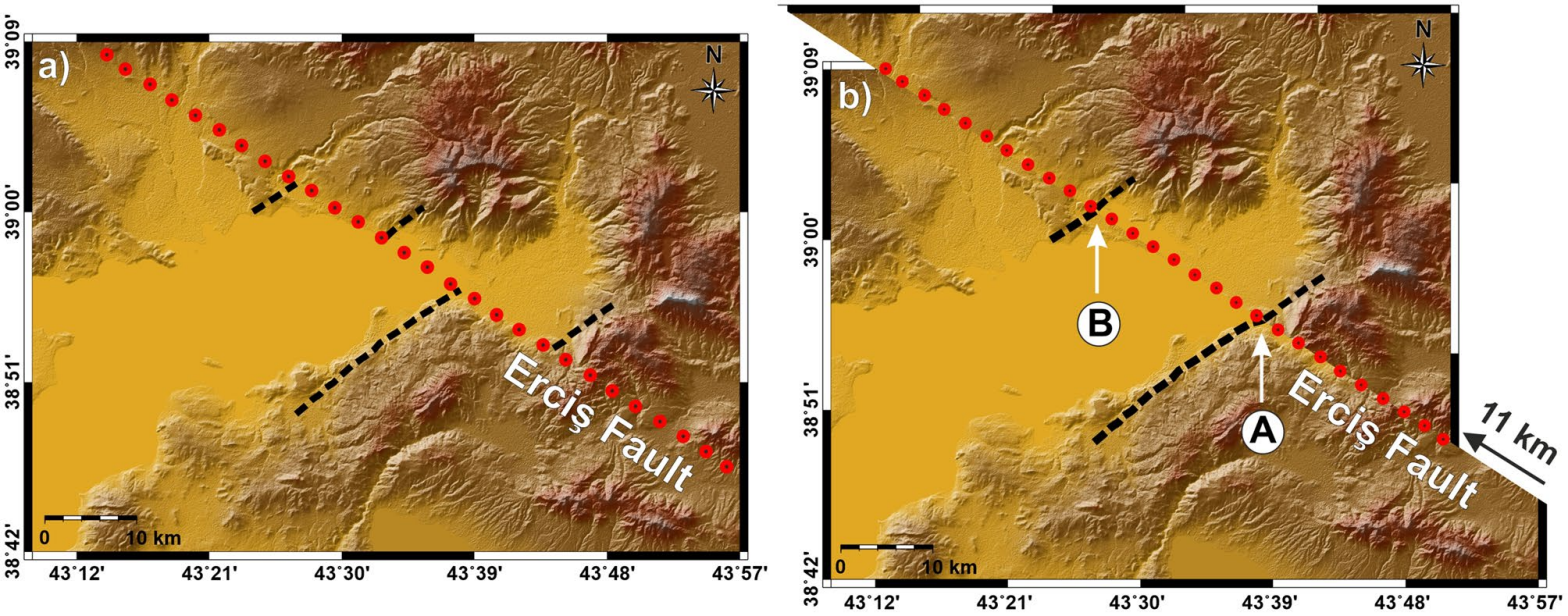
Restoration of 11 km movement on Erciş Fault. (**a**) Present state of the area after 11 kms of tectonic shift. (**b**) The state of the area before the tectonic shift, restored by reversing the process visually. Red dotted line represents Erciş Fault and the dashed black lines represent mountain fronts marked in^80^. Maps were created by using the Generic Mapping Tools software, version 5.1.1. (https://www.soest.hawaii.edu/gmt/)^84^.

Copley and Jackson^80^, investigating the active tectonics of the Turkish–Iranian plateau, examined the right lateral strike-slip faults and claimed that the parallel striking Erciş Fault and Çaldıran Fault are 11 km and 1.3 km offset, respectively. Furthermore, they stated that the combined slip rate for these faults was about 8 mm/year and that a time of about 1.5 Ma would be necessary to generate the combined 12.3 km shift at this speed. Authors stated that the removal of 11 km of dextral movement along the Erciş Fault restores the mountain-fronts (Fig. 8a) and the edge of volcanic rocks (Fig. 8b).

Koçyiğit^81^ claimed that the Erciş Fault cut across Early-Middle Miocene marine sequence and Quaternary volcanic rocks to sedimentary packages, also Çakırbey Fault cuts across the Early–Middle Miocene marine limestone, Quaternary volcanic rocks. However, as it is seen from our new paleomagnetic rotations, the northern part of the older Çakırbey Fault was cut and shifted by Erciş Fault. This displacement caused the oppositely directed rotations around Muradiye.

**Figure 9:**
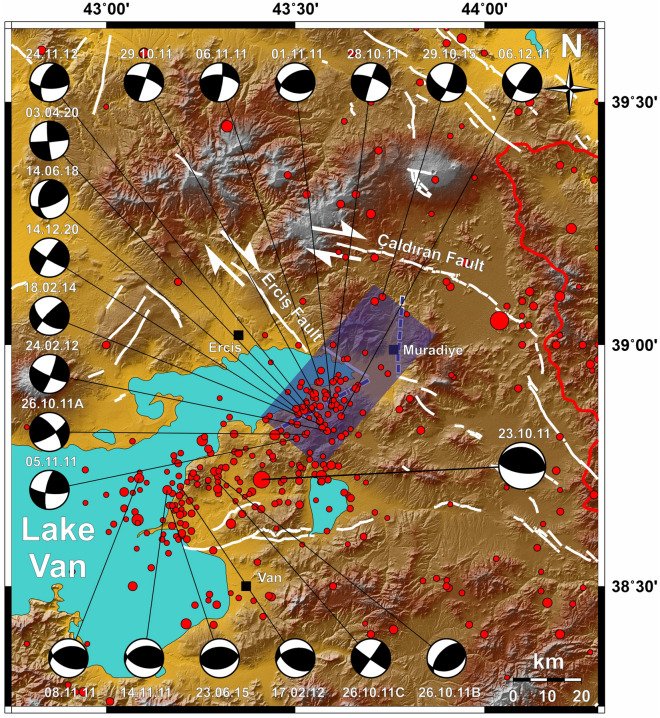
Seismicity of the Lake Van and the surrounding area. M > 4.0 earthquakes between 01.01.1900–21.02.2021. Earthquake epicenter data are retrieved from Kandilli Observatory and Earthquake Research Institute (KOERI) and focal mechanism solutions of M > 4.5 earthquakes between 23.10.2011 (after the 23 October 2011, M = 7.2)—21.02.2021 from “Disaster and Emergency Management Authority of Turkey (AFAD) Presidential of Earthquake Department” Fault Mechanism system. Only focal mechanism solutions of the earthquakes on the possible Çakırbey Fault and 23 October 2011 Van earthquake were drawn. (dashed blue line represents possible Çakırbey Fault). Map was created by using the Generic Mapping Tools software, version 5.1.1. (https://www.soest.hawaii.edu/gmt/)^84^.

In Fig. 9, the focal mechanism distributions of M > 4.5 earthquakes on the possible Çakırbey Fault are given on the northeastern border of Lake Van. Also, Koçyiğit^81^ shows that the Eastern plateau is under the influence of the N-S direction of the Arabian plate by using GPS data and focal mechanism solutions of different earthquakes. Our results and the distribution and the focal mechanisms of earthquakes are examined, it is clear that a left lateral strike slip Çakırbey fault exists and is active.

On the northern southern parts of the Erciş Fault, ~ 13° clockwise and ~ 1° counterclockwise rotations are seen, respectively, between Pliocene–Pleistocene time intervals. These rotations indicate a deformation around a right lateral strike-slip fault and the block movements on both sides of the fault. In summary, according to the results obtained from this study, the study area had a rotation of ~ 2° counterclockwise in the Late Miocene–Pliocene time interval. It was rotated clockwise ~ 13° in the Pliocene–Pleistocene period. Since the Pleistocene, it has rotated ~ 14° counterclockwise.

## Conclusions

Widespread and intense seismic activity, rapid uplift of the Eastern Anatolian plateau, westward movement of the Anatolian plate along the NAFZ and EAFZ, strike-slip fault zones in the region and Neogene magmatic activity throughout the region are indicators of the region's complex tectonic structure. At the same time, the fact that the paleomagnetic rotations obtained from our study using the same aged rock groups scattered in different regions is another indicator of the high tectonic activity of the region.

In this study, 62 reliable paleomagnetic sites were taken from the Late Miocene to Quaternary volcanic rocks located to the north of Lake Van, Eastern Anatolia, in order to examine the tectonic deformation of the north of the Lake Van from a paleomagnetic aspect. Hisarlı et al.^16^ stated that the region moved as a single block rotating clockwise by using a limited number of paleomagnetic sites sparsely covering the study area. By increasing the number and distribution of paleomagnetic sites, our study revealed smaller blocks that are moving in different directions, both clockwise and counter clockwise in the area.

When the inclination angles of the group mean values given in Table 2 are considered, the value is very close (with 2–3° difference) to the expected inclination angle value (58°) for the region, which is a sign that the region has not been making a latitudinal movement since the Late Miocene.

In the vicinity of the western part of Erciş Fault, rotations are observed in all age groups in the Late Miocene–Pleistocene time interval depending on the activity of the Erciş fault. Also, paleomagnetic data from the Pliocene volcanics on the north-western part of Erçek Lake, Van-Muradiye highway (around Çolpan village near the Lake Van) defined R ± ΔR = 24.4 ± 17.0 clockwise rotation. However, there is no active fault in this area on the new active fault map of Turkey^78,79^. These paleomagnetic rotations and focal mechanism solutions of the recent earthquakes in the field indicate that the former Çakırbey Fault might still be active.

## Supplementary Information

Supplementary Information.

## Data Availability

All the data achieved by the analysis of the collected samples are given in tables with the manuscript.

## Abbreviations

AFAD: Disaster and Emergency Management Authority of Turkey
CCW: Counterclockwise
ChRM: Characteristic remanent magnetization
EAFZ: East Anatolian fault zone
EKP: Erzurum-Kars plateau
HTS: High temperature susceptibility
IRM: Isothermal remanent magnetization
ITU: Istanbul Technical University
NAFZ: North Anatolian fault zone
NRM: Natural remanent magnetization
PSV: Paleosecular variations
VB: Van block
VGP: Virtual geomagnetic pole
