# Supplementary material for: New paleomagnetic results from Neogene to Quaternary volcanic rocks of north of the Lake Van, Eastern Turkey
Source: Sci Rep. 2023 Jul 27;13:12206. doi: 10.1038/s41598-023-39492-w (PMC10374621; doi:10.1038/s41598-023-39492-w)
Supplement: Supplementary file 1 — Supplementary Information. [file 41598_2023_39492_MOESM1_ESM.zip › Supplementary_Table_A1_2023.docx]

**Table A1.** Neogene to Quaternary time interval site and group mean paleomagnetic results from North of the Lake Van, Eastern Anatolia, N: number of demagnetized specimens, **S:** VGP dispersion, **Sl / Su**: upper / lower 95 % confidence limits of S [52], **D/I**: declination / inclination, **ΔDx / (ΔIx)**: errors in declination / inclination determined from the A95 of the poles, **k**: estimate of the precision parameter determined from the ChRM directions, **α_95_**: cone of confidence determined from the mean ChRM directions, **K** : precision parameter determined from the mean virtual geomagnetic pole directions (VGPs), **A95**: cone of confidence determined from the mean VGP direction, **A95min/A95max**: minimum / maximum value of the A95 [52], **λ**: paleolatitude. **Pliocene_SW**: Group means of Pliocene aged sites located in the Southwestern part of the Erciş Fault, **Pliocene_NE**: Group means of Pliocene aged sites located in the Northeastern part of the Erciş Fault, **Pliocene_EV:** Group means of Pliocene aged sites located in the Eastern part of the Erciş Fault and Near the Van, **Pliocene_EM:** Group means of Pliocene aged sites located in the Eastern part of the Erciş Fault and Near the Muradiye. ** Sites with A95 values outside the envelope of A95min and A95max.

Pleistocene

| **Site** | **N** | **Sl** | **S** | **Su** | **D** | **ΔDx** | **I** | **ΔIx** | **R** | **k** | **α_95_** | **K** | **A95** | **A95_min_** | **A95_max_** | **λ** |
| --- | --- | --- | --- | --- | --- | --- | --- | --- | --- | --- | --- | --- | --- | --- | --- | --- |
| aci123 | 18 | 14,3 | 17,1 | 20,5 | 344,5 | 8,5 | 47,6 | 8,8 | 17,37 | 22,7 | 6,8 | 22,7 | 7,4 | 3,8 | 13,3 | 28,7 |
| bend | 9 | 7,2 | 9,7 | 12,5 | 356,1 | 7,7 | 55,9 | 6 | 8,921 | 101,9 | 5,1 | 69,6 | 6,2 | 5 | 20,5 | 36,5 |
| **ckk2**** | 8 | 3,1 | 5,1 | 6,2 | 355,9 | 3,9 | 44,2 | 4,4 | 7,98 | 345,3 | 3 | 256,2 | 3,5 | 5,2 | 22,1 | 25,9 |
| dlcy | 7 | 9,8 | 14,7 | 18,3 | 354,3 | 13,7 | 55,4 | 10,9 | 6,877 | 48,7 | 8,7 | 30,5 | 11,1 | 5,5 | 24,1 | 35,9 |
| colp12 | 14 | 12,5 | 17,5 | 21,8 | 338,4 | 8,8 | 12,7 | 16,9 | 13,287 | 18,2 | 9,6 | 21,6 | 8,7 | 4,2 | 15,6 | 6,4 |
| hac3 | 8 | 7,1 | 11,4 | 13,2 | 351,8 | 9,1 | 49,1 | 9 | 7,905 | 73,6 | 6,5 | 50,8 | 7,8 | 5,2 | 22,1 | 30 |
| inc12 | 12 | 10 | 13,6 | 17,5 | 345,4 | 8,6 | 50,6 | 8,1 | 11,764 | 46,7 | 7,3 | 36 | 7,3 | 4,4 | 17,1 | 31,4 |
| kek12 | 13 | 13,4 | 17,5 | 22,6 | 333,7 | 10,3 | 45,7 | 11,2 | 12,604 | 30,3 | 7,7 | 21,6 | 9,1 | 4,3 | 16,3 | 27,1 |
| koz5 | 7 | 5,7 | 10,6 | 15,4 | 55,9 | 8,1 | 25,0 | 13,7 | 6,905 | 63,1 | 7,7 | 59,0 | 7,9 | 5,5 | 24,1 | 13,1 |
| inc4 | 7 | 6,6 | 9,9 | 12,0 | 47,5 | 7,9 | 38,1 | 10,6 | 6,902 | 61,3 | 7,8 | 67,7 | 7,4 | 5,5 | 24,1 | 21,4 |
| **kad2**** | 5 | 1,4 | 2,3 | 1,9 | 0,5 | 2,4 | 44,0 | 2,8 | 4,997 | 1148 | 2,3 | 1214,6 | 2,2 | 6,3 | 29,7 | 25,8 |
| oya | 8 | 5,4 | 7,9 | 9,1 | 342,8 | 6,3 | 48,4 | 6,3 | 7,929 | 98 | 5,6 | 104,5 | 5,4 | 5,2 | 22,1 | 29,4 |
| arg | 7 | 4,7 | 9,6 | 11,1 | 358,2 | 8,2 | 48,5 | 8,3 | 6,941 | 101,6 | 6 | 71,9 | 7,2 | 5,5 | 24,1 | 29,5 |
| tprk67 | 17 | 10,7 | 14,5 | 17,3 | 347,6 | 7,5 | 49,9 | 7,2 | 16,508 | 32,5 | 6,3 | 31,7 | 6,4 | 3,9 | 13,8 | 30,7 |
| ykr | 7 | 12,8 | 16,3 | 21,8 | 359 | 14,6 | 51,6 | 13,3 | 6,831 | 35,6 | 10,3 | 24,8 | 12,4 | 5,5 | 24,1 | 32,2 |
| yln | 7 | 9,3 | 16,7 | 21,1 | 336,9 | 14,4 | 47,4 | 15 | 6,776 | 26,7 | 11,9 | 23,8 | 12,6 | 5,5 | 24,1 | 28,5 |
| **Pleistocene** | **127/114** | **14,8** | **16,7** | **18,4** | **347,5** | **3,2** | **49,9** | **3,1** | **110,055** | **31,8** | **2,4** | **24,3** | **2,8** | **1,8** | **4,2** | **30,7** |

Pliocene_SW of Erciş Fault

| **Site** | **N** | **Sl** | **S** | **Su** | **D** | **ΔDx** | **I** | **ΔIx** | **R** | **k** | **α_95_** | **K** | **A95** | **A95_min_** | **A95_max_** | **λ** |
| --- | --- | --- | --- | --- | --- | --- | --- | --- | --- | --- | --- | --- | --- | --- | --- | --- |
| **ako**** | 7 | 4,2 | 6 | 8,2 | 195,5 | 6,9 | -66,8 | 3,3 | 6,987 | 455,9 | 2,8 | 183,9 | 4,5 | 5,5 | 24,1 | 49,3 |
| atok12 | 13 | 8,0 | 12 | 16,3 | 1,2 | 9,4 | 66,5 | 4,6 | 12,876 | 45,9 | 4,2 | 45,9 | 6,2 | 4,3 | 16,3 | 49 |
| bend2 | 8 | 6 | 8,7 | 10,3 | 192,9 | 7,7 | -57,7 | 5,6 | 7,947 | 131,3 | 4,9 | 86,1 | 6 | 5,2 | 22,1 | 38,4 |
| kaditvt | 13 | 5,5 | 8,5 | 10,4 | 177,9 | 5,4 | -54,9 | 4,4 | 12,908 | 130,9 | 3,6 | 90,6 | 4,4 | 4,3 | 16,3 | 35,5 |
| koc | 6 | 7,1 | 10,5 | 14,8 | 173,6 | 9,8 | -45,2 | 10,8 | 5,905 | 52,4 | 9,3 | 60,1 | 8,7 | 5,9 | 26,5 | 26,7 |
| krh12 | 16 | 13,5 | 15,6 | 17,9 | 158,1 | 11,5 | -68,1 | 5,1 | 15,788 | 70,8 | 4,4 | 27,3 | 7,2 | 4,0 | 14,3 | 51,2 |
| kzcukg | 15 | 9,8 | 12,4 | 15,8 | 172,9 | 8,0 | -61,4 | 5,0 | 18,415 | 75,6 | 4,4 | 43,4 | 5,9 | 4,1 | 14,9 | 42,5 |
| trtptrant | 22 | 13,6 | 16,9 | 20,3 | 184,2 | 8,4 | -58,0 | 6,0 | 21,465 | 39,2 | 5,0 | 23,4 | 6,6 | 3,5 | 11,7 | 38,7 |
| san12shl | 24 | 11,2 | 12,9 | 14,8 | 145,1 | 6,6 | -62,2 | 3,9 | 23,688 | 73,7 | 3,5 | 39,6 | 4,8 | 3,4 | 11,1 | 43,5 |
| skr | 8 | 5,2 | 9,2 | 12,4 | 151,1 | 7,8 | -55,5 | 6,2 | 7,906 | 74,6 | 6,5 | 78,4 | 6,3 | 5,2 | 22,1 | 36 |
| snty | 8 | 4,8 | 8,5 | 9,2 | 170,6 | 10,1 | -70,3 | 3,9 | 7,964 | 193,1 | 4,0 | 91,2 | 5,8 | 5,2 | 22,1 | 54,4 |
| **kisla2**** | 6 | 2,7 | 3,4 | 3,9 | 182,5 | 2,9 | -28,7 | 4,6 | 5,987 | 380,4 | 3,4 | 582,3 | 2,8 | 5,9 | 26,5 | 15,3 |
| tas | 8 | 5,3 | 8,0 | 8,8 | 165,1 | 7,1 | -58,7 | 5,0 | 7,960 | 174,2 | 4,2 | 101,8 | 5,5 | 5,2 | 22,1 | 39,4 |
| uns12 | 15 | 6,3 | 8,8 | 10,5 | 162,0 | 6,0 | -64,1 | 3,3 | 14,911 | 84,5 | 3,1 | 84,5 | 4,2 | 4,1 | 14,9 | 45,8 |
| ytokisik | 15 | 8,4 | 10,8 | 13,5 | 159,6 | 6,3 | -54,4 | 5,2 | 14,853 | 95,4 | 3,9 | 56,5 | 5,1 | 4,1 | 14,9 | 34,9 |
| kzc2 | 7 | 6,4 | 7,4 | 9,5 | 171,8 | 6,9 | -56,2 | 5,3 | 6,964 | 166,8 | 4,7 | 121,1 | 5,5 | 5,5 | 24,1 | 36,7 |
| hydr3 | 8 | 13 | 19 | 26,1 | 126,3 | 21,8 | -68,7 | 9,2 | 7,846 | 45,4 | 8,3 | 18,6 | 13,2 | 5,2 | 22,1 | 52,1 |
| tel | 8 | 3,3 | 7,7 | 10,4 | 164,5 | 6,3 | -53,2 | 5,5 | 7,954 | 150,7 | 4,5 | 111,5 | 5,3 | 5,2 | 22,1 | 33,8 |
| **Pliocene_SW** | **194/192** | **16,6** | **17,9** | **19,2** | **346,9** | **3,1** | **60,9** | **1,9** | **187,474** | **39,1** | **1,6** | **21,0** | **2,3** | **1,5** | **3,0** | **41,9** |

Pliocene_Near Van

| **Site** | **N** | **Sl** | **S** | **Su** | **D** | **ΔDx** | **I** | **ΔIx** | **R** | **k** | **α_95_** | **K** | **A95** | **A95_min_** | **A95_max_** | **λ** |
| --- | --- | --- | --- | --- | --- | --- | --- | --- | --- | --- | --- | --- | --- | --- | --- | --- |
| **nors**** | 9 | 2,3 | 4,6 | 6,5 | 44,8 | 3,6 | 53,8 | 3 | 8,982 | 437,7 | 2,5 | 304,1 | 3 | 5 | 20,5 | 34,3 |
| sglm | 5 | 14 | 26,6 | 30,3 | 37,4 | 38,6 | 63,4 | 20,9 | 4,77 | 17,4 | 18,9 | 9,5 | 26,2 | 6,3 | 29,7 | 45 |
| yayla12 | 14 | 11,4 | 17,3 | 23,1 | 23,6 | 15,8 | 71,8 | 5,6 | 13,802 | 65,7 | 4,9 | 22,2 | 8,6 | 4,2 | 15,6 | 56,7 |
| timar_tilt | 6 | 6 | 8,8 | 10,7 | 20,9 | 9,5 | 59 | 6,5 | 5,966 | 146,9 | 5,5 | 85,5 | 7,3 | 5,9 | 26,5 | 39,8 |
| **Pliocene_EV** | **25/25** | **15,0** | **18,7** | **22,4** | **25,3** | **16,0** | **66,9** | **5,0** | **24,061** | **41,7** | **4,7** | **19,8** | **6,8** | **3,3** | **10,9** | **49,5** |

Pliocene_Around_Muradiye

| **Site** | **N** | **Sl** | **S** | **Su** | **D** | **ΔDx** | **I** | **ΔIx** | **R** | **k** | **α_95_** | **K** | **A95** | **A95_min_** | **A95_max_** | **λ** |
| --- | --- | --- | --- | --- | --- | --- | --- | --- | --- | --- | --- | --- | --- | --- | --- | --- |
| bbc12 | 16 | 8,4 | 14,4 | 16,8 | 208,3 | 8,4 | -57,5 | 6,2 | 15,615 | 39 | 6,0 | 32,2 | 6,6 | 4,0 | 14,3 | 38,1 |
| blkbrj | 14 | 11,7 | 14,6 | 17,8 | 171,0 | 8,7 | -53,3 | 7,5 | 13,738 | 49,7 | 5,7 | 31,3 | 7,2 | 4,2 | 15,6 | 33,8 |
| dere | 8 | 5,9 | 9,5 | 13,6 | 9,7 | 6,8 | 32,6 | 10,2 | 7,894 | 66,3 | 6,9 | 73,1 | 6,5 | 5,2 | 22,1 | 17,7 |
| drk12 | 17 | 7,8 | 10,4 | 11,1 | 180,9 | 5,5 | -53,0 | 4,8 | 16,806 | 82,6 | 3,9 | 61,1 | 4,6 | 3,9 | 13,8 | 33,6 |
| kmr12 | 16 | 8,1 | 9,8 | 10,6 | 188,1 | 5,0 | -43,1 | 5,8 | 15,688 | 48,1 | 5,4 | 68,2 | 4,5 | 4,0 | 14,3 | 25,1 |
| kmr3mur_tilt | 15 | 11,5 | 15,1 | 18,6 | 192,2 | 7,7 | -35,0 | 10,9 | 14,541 | 30,5 | 7,0 | 28,9 | 7,2 | 4,1 | 14,9 | 19,3 |
| **pliocene_EM** | **86/85** | **15,7** | **17,7** | **19,6** | **8,1** | **3,9** | **47,5** | **4,0** | **81,816** | **24,8** | **3,2** | **21,5** | **3,4** | **2,0** | **5,0** | **28,6** |

Pliocene NE of Erciş Fault

| **Site** | **N** | **Sl** | **S** | **Su** | **D** | **ΔDx** | **I** | **ΔIx** | **R** | **k** | **α_95_** | **K** | **A95** | **A95_min_** | **A95_max_** | **λ** |
| --- | --- | --- | --- | --- | --- | --- | --- | --- | --- | --- | --- | --- | --- | --- | --- | --- |
| al127 | 20 | 18,8 | 21,5 | 24,3 | 176,9 | 10,7 | -52,7 | 9,4 | 19,076 | 20,6 | 7,4 | 14,4 | 8,9 | 3,6 | 12,4 | 33,3 |
| al3 | 12 | 7,6 | 9,6 | 12,2 | 173,6 | 6,1 | -51,4 | 5,6 | 11,895 | 104,5 | 4,3 | 71,8 | 5,2 | 4,4 | 17,1 | 32 |
| goz124 | 21 | 14,2 | 17,9 | 22,0 | 167,8 | 8,4 | -51,3 | 7,7 | 20,309 | 29 | 6,0 | 20,9 | 7,1 | 3,6 | 12 | 32 |
| goz5 | 6 | 7,2 | 12,6 | 18,8 | 344,4 | 12,3 | 50,5 | 11,7 | 5,897 | 48,5 | 9,7 | 41,4 | 10,5 | 5,9 | 26,5 | 31,2 |
| koz2 | 6 | 14,1 | 17,8 | 19,7 | 13,1 | 16,4 | 41 | 20,3 | 5,775 | 22,2 | 14,5 | 20,9 | 15 | 5,9 | 26,5 | 23,5 |
| koz6 | 7 | 4,7 | 8,4 | 11,4 | 184,3 | 7,5 | -52,4 | 6,6 | 6,957 | 140,5 | 5,1 | 94 | 6,3 | 5,5 | 24,1 | 33 |
| pay2 | 6 | 12 | 17,5 | 24,2 | 16,9 | 17,6 | 52,5 | 15,5 | 5,797 | 24,6 | 13,8 | 21,7 | 14,7 | 5,9 | 26,5 | 33,1 |
| snty2 | 8 | 5,6 | 8,5 | 7,9 | 131,2 | 10,3 | -70,7 | 3,9 | 7,964 | 196,3 | 4,0 | 90,1 | 5,9 | 5,2 | 22,1 | 55,0 |
| tprkykoz | 13 | 11,3 | 15,5 | 20,3 | 171,8 | 10,5 | -59,2 | 7,2 | 12,724 | 43,4 | 6,4 | 27,7 | 8,0 | 4,3 | 16,3 | 40,0 |
| tprk3 | 7 | 10,2 | 18,8 | 25,3 | 205,2 | 17 | -51,8 | 15,3 | 6,804 | 30,6 | 11,1 | 18,8 | 14,3 | 5,5 | 24,1 | 32,5 |
| ykoz35 | 13 | 14,1 | 17,2 | 19,6 | 196,2 | 11,0 | -55,1 | 8,9 | 12,713 | 41,8 | 6,5 | 22,5 | 8,9 | 4,3 | 16,3 | 35,6 |
| ykoz4 | 8 | 8,0 | 11,9 | 16,3 | 168,9 | 9,7 | -51,3 | 8,9 | 7,902 | 71,3 | 6,6 | 46,4 | 8,2 | 5,2 | 22,1 | 32,0 |
| tprk24 | 17 | 9,5 | 12,4 | 14,1 | 215,5 | 7,5 | -61,3 | 4,7 | 16,827 | 92,7 | 3,7 | 42,8 | 5,5 | 3,9 | 13,8 | 42,4 |
| goz3 | 9 | 3,0 | 9,9 | 10,1 | 162,3 | 7,3 | -48,7 | 7,3 | 8,91 | 88,8 | 5,5 | 67,7 | 6,3 | 5,0 | 20,5 | 29,7 |
| ykoz2 | 8 | 8,5 | 13,8 | 17,4 | 224,1 | 16,3 | -69,8 | 6,5 | 7,91 | 78,1 | 6,3 | 34,6 | 9,5 | 5,2 | 22,1 | 53,7 |
| **koz7**** | 8 | 2,6 | 3,6 | 4,7 | 198,9 | 3 | -53,5 | 2,6 | 7,991 | 811,4 | 1,9 | 501,7 | 2,5 | 5,2 | 22,1 | 34,1 |
| al4 | 7 | 5,1 | 9,8 | 12 | 163,8 | 8,8 | -52,5 | 7,8 | 6,92 | 75,1 | 7 | 68,4 | 7,3 | 5,5 | 24,1 | 33,1 |
| hydr12 ykoz4 | 23 | 14,4 | 20,2 | 24,6 | 164,8 | 9,7 | -57,1 | 7,2 | 22,287 | 30,9 | 5,5 | 16,5 | 7,7 | 3,4 | 11,4 | 37,7 |
| **Pliocene_NE** | **183/179** | **20,5** | **22,1** | **23,5** | **359,9** | **3,7** | **56,3** | **2,8** | **170,752** | **22,7** | **2,3** | **13,9** | **2,9** | **1,5** | **3,1** | **36,6** |

Late Miocene

| **Site** | **N** | **Sl** | **S** | **Su** | **D** | **ΔDx** | **I** | **ΔIx** | **R** | **k** | **α_95_** | **K** | **A95** | **A95_min_** | **A95_max_** | **λ** |
| --- | --- | --- | --- | --- | --- | --- | --- | --- | --- | --- | --- | --- | --- | --- | --- | --- |
| ack2 | 7 | 5,9 | 8,7 | 11,1 | 195,6 | 7,8 | -53,6 | 6,7 | 6,955 | 133,7 | 5,2 | 87,8 | 6,5 | 5,5 | 24,1 | 34,2 |
| **cay23**** | 16 | 4,2 | 5,6 | 7,4 | 189,9 | 3,1 | -54,0 | 3,1 | 15,956 | 342,6 | 2,0 | 211,7 | 2,5 | 4,0 | 14,3 | 34,5 |
| **cay4**** | 8 | 3,2 | 4,1 | 5,3 | 192,1 | 3,6 | -57 | 2,7 | 7,99 | 713,7 | 2,1 | 386,6 | 2,8 | 5,2 | 22,1 | 37,6 |
| **haci**** | 8 | 3,5 | 4,5 | 5,4 | 338,8 | 3,8 | 55,7 | 3 | 7,985 | 470,5 | 2,6 | 331,7 | 3 | 5,2 | 22,1 | 36,3 |
| hclykr2 | 17 | 5,6 | 8,3 | 8,6 | 157,0 | 4,6 | -50,0 | 4,4 | 16,857 | 111,9 | 3,4 | 82,0 | 4,0 | 3,9 | 13,8 | 30,8 |
| orn | 7 | 5,6 | 10,3 | 14 | 39,9 | 10,5 | 61,6 | 6,5 | 6,948 | 114,8 | 5,7 | 62,5 | 7,7 | 5,5 | 24,1 | 42,7 |
| payack | 16 | 13,2 | 16,0 | 17,9 | 192,4 | 9,4 | -57,0 | 7,0 | 15,706 | 51,1 | 5,2 | 25,8 | 7,4 | 4,0 | 14,3 | 37,6 |
| yln2kiz2 | 11 | 17,0 | 22,0 | 28,5 | 165,4 | 13,9 | -40,4 | 17,4 | 10,472 | 18,9 | 10,8 | 13,8 | 12,7 | 4,6 | 18,1 | 23 |
| kiz | 7 | 6,4 | 8,9 | 11,2 | 166,2 | 7,8 | -49,4 | 7,6 | 6,924 | 79 | 6,8 | 82,3 | 6,7 | 5,5 | 24,1 | 30,2 |
| **Late Miocene** | **65/64** | **18,8** | **21,1** | **23,4** | **341,7** | **5,7** | **53,2** | **4,9** | **61,184** | **22,8** | **3,8** | **15,2** | **4,7** | **2,3** | **6,0** | **33,7** |
