# Supplementary figures and images for: New paleomagnetic results from Neogene to Quaternary volcanic rocks of north of the Lake Van, Eastern Turkey
Source: Sci Rep. 2023 Jul 27;13:12206. doi: 10.1038/s41598-023-39492-w (PMC10374621; doi:10.1038/s41598-023-39492-w)

Figure A1. Normalized IRM acquisition curves


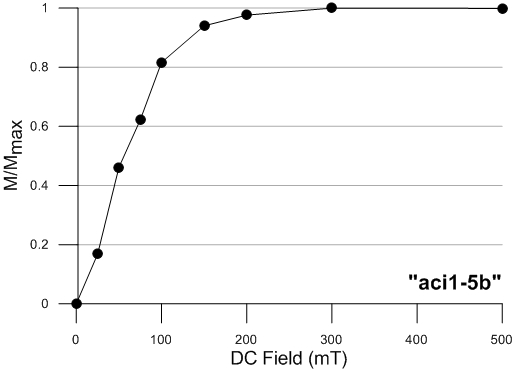

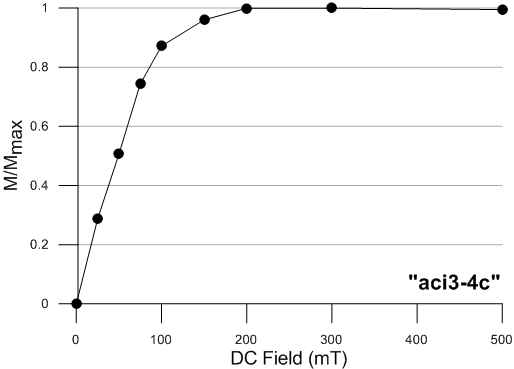


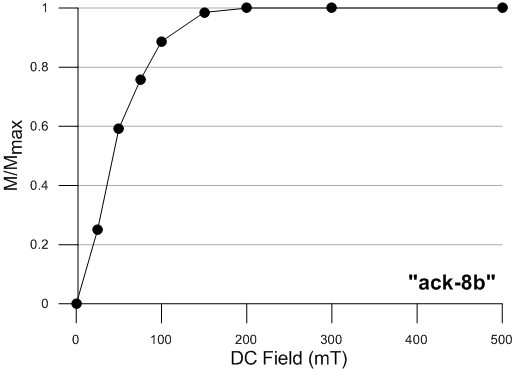

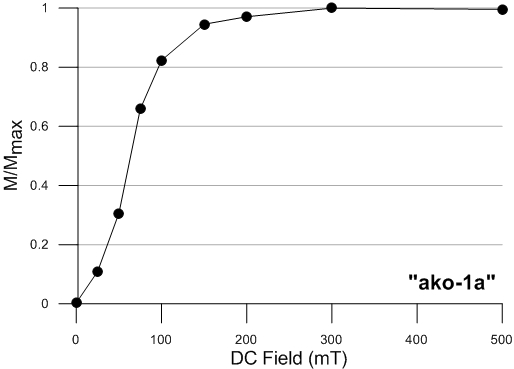


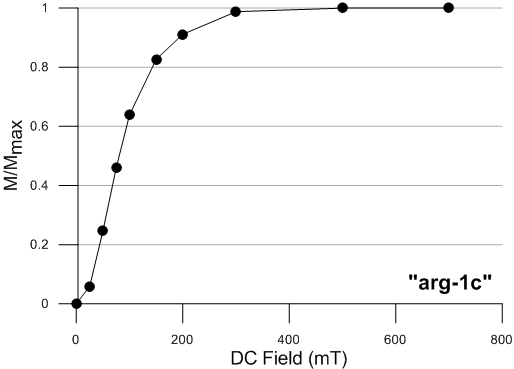

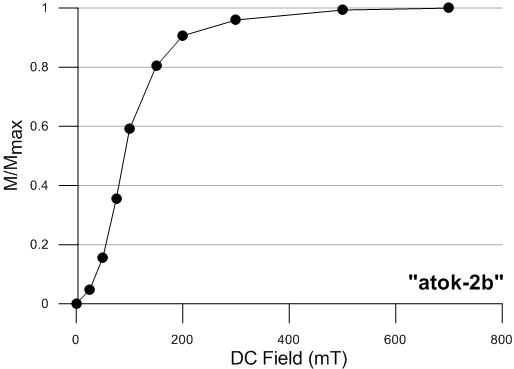


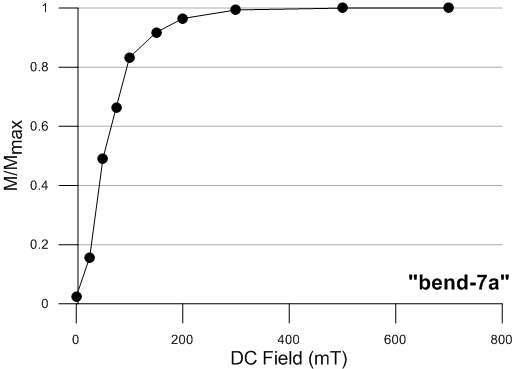

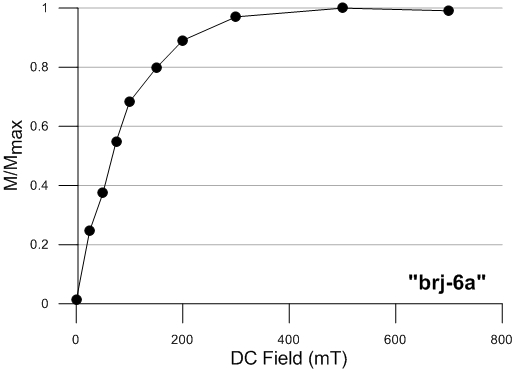


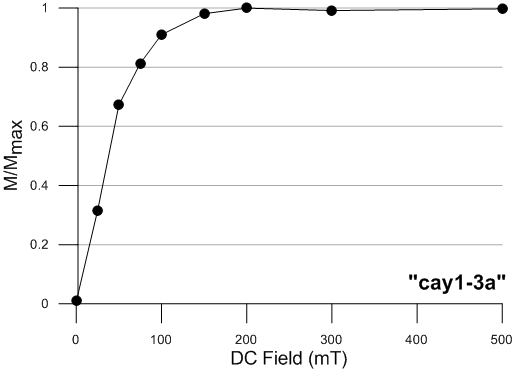

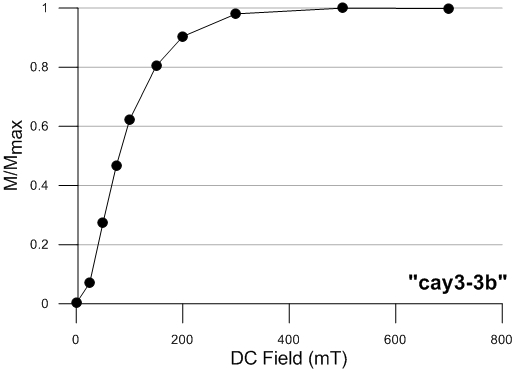


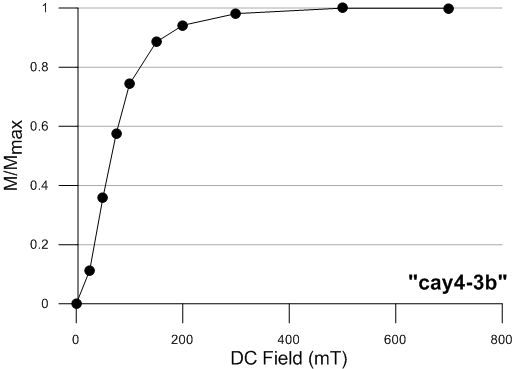

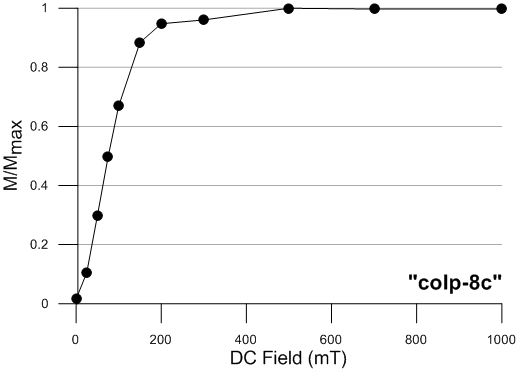


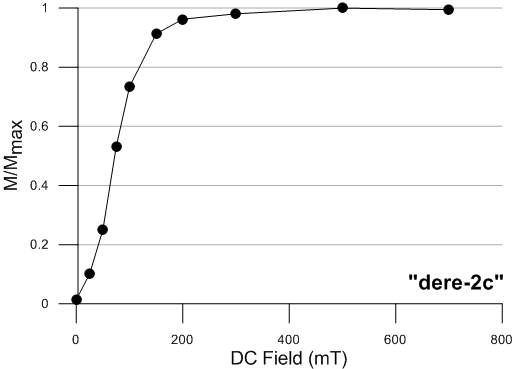

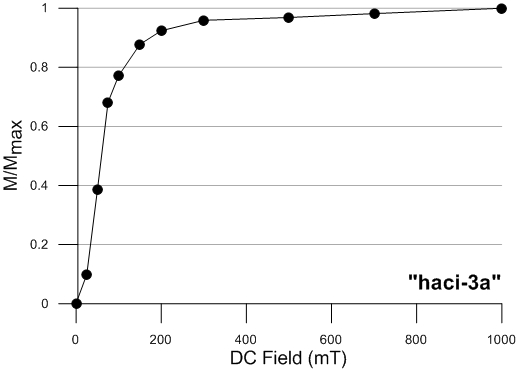


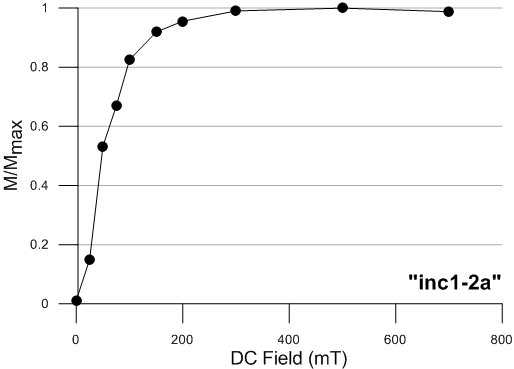

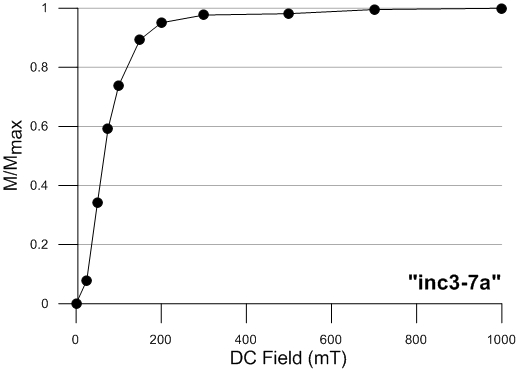


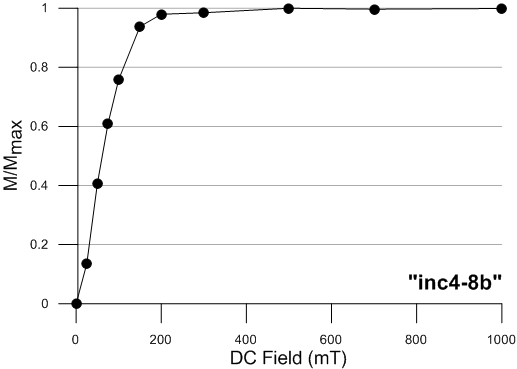

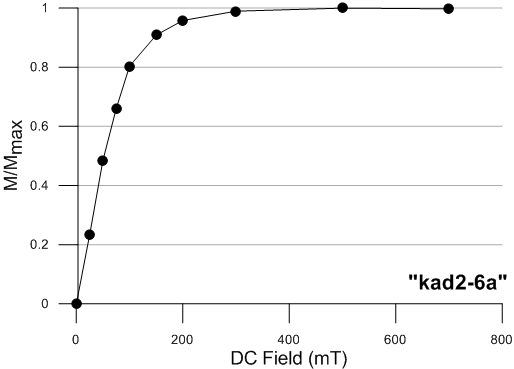


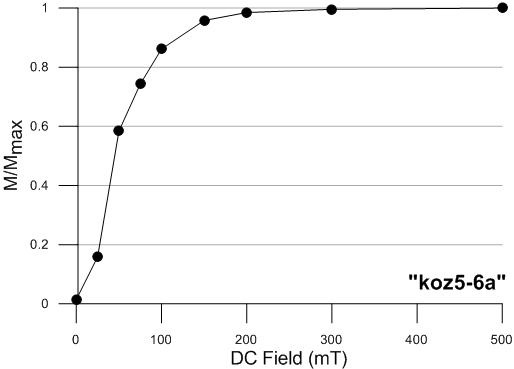

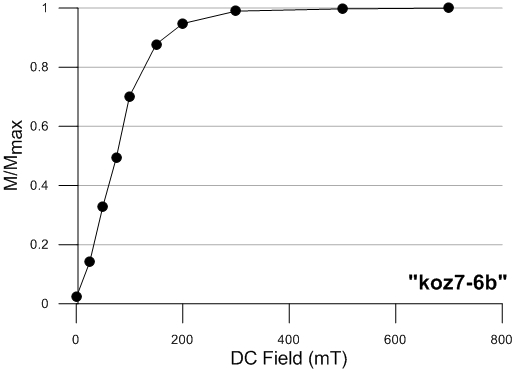


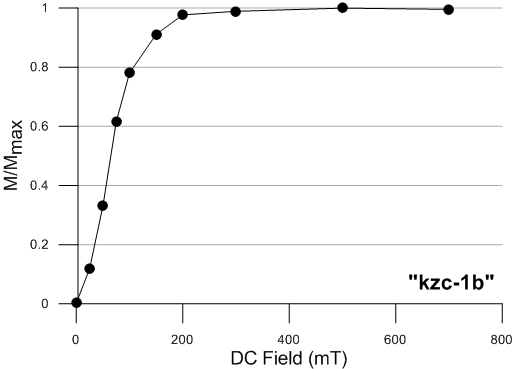

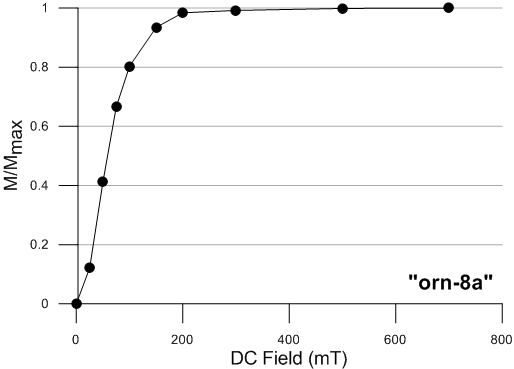


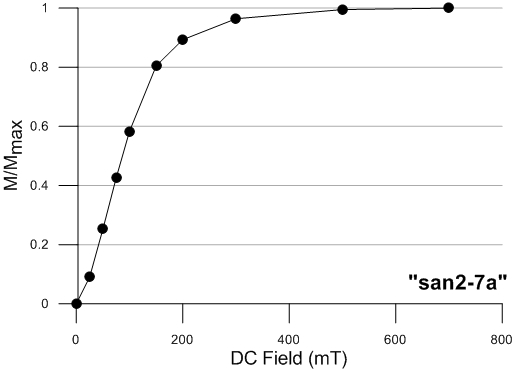

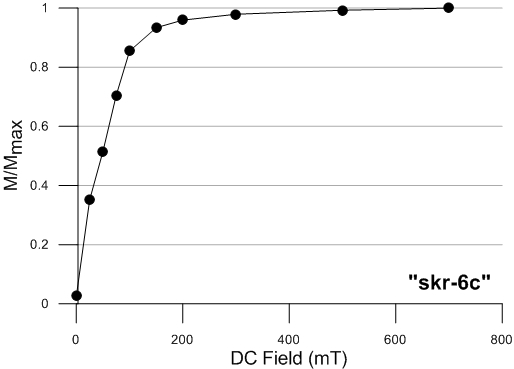

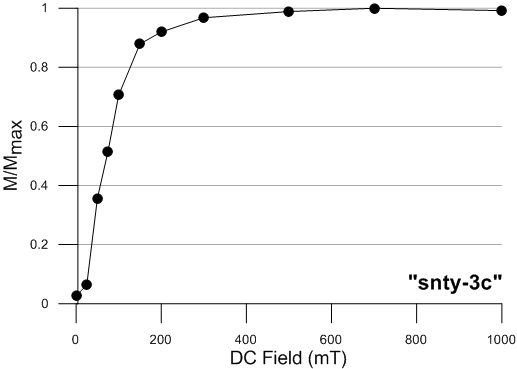

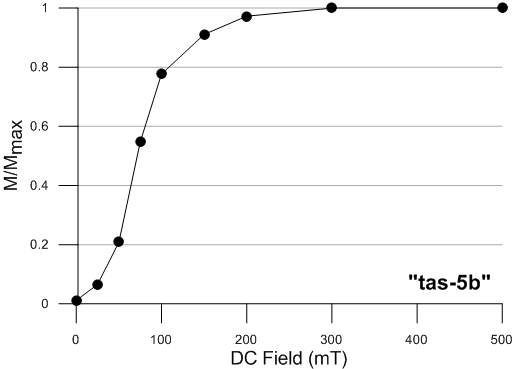


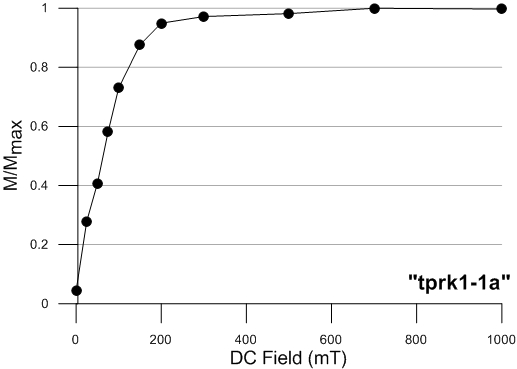

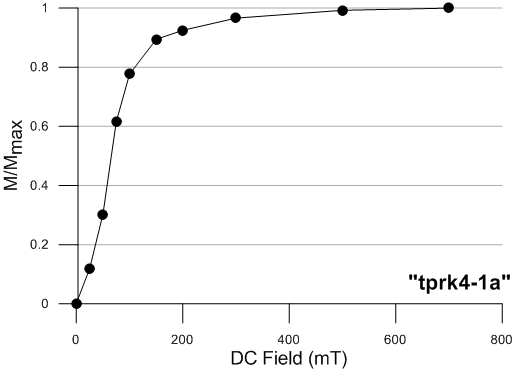


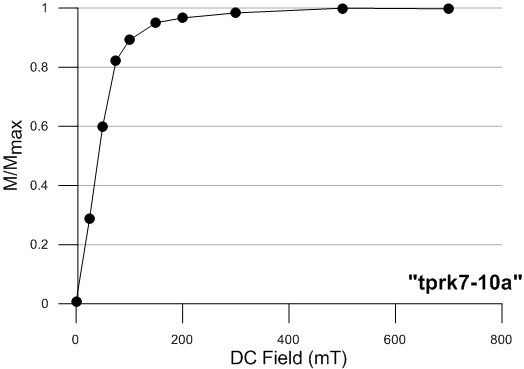

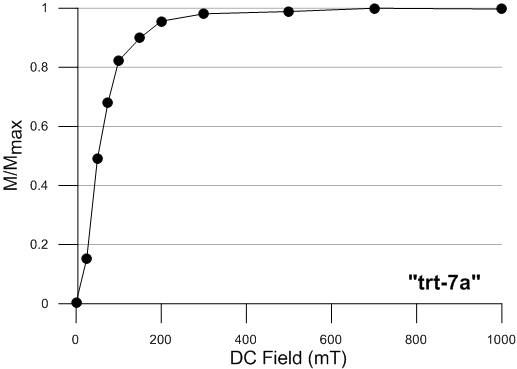


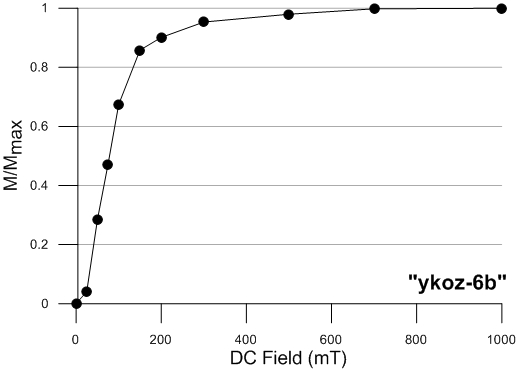

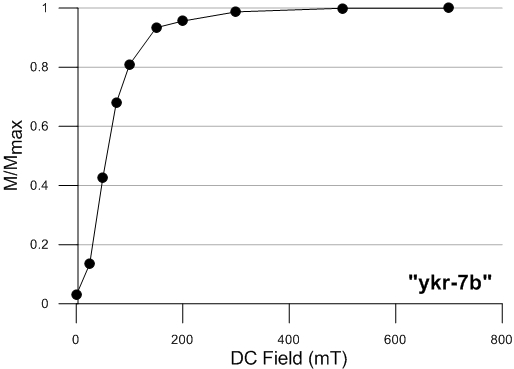

Supplement: Supplementary file 1 — Supplementary Information. [file 41598_2023_39492_MOESM1_ESM.zip › Supplementary_Figure_A1.docx]

Figure A2. High temperature susceptibility curves


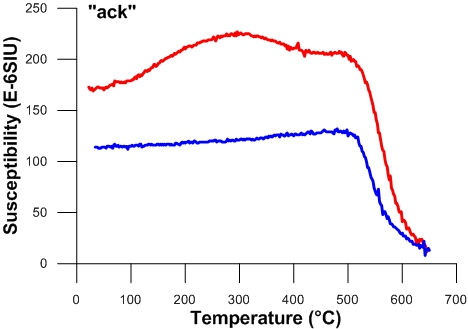

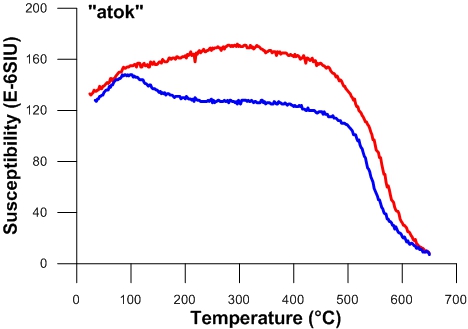


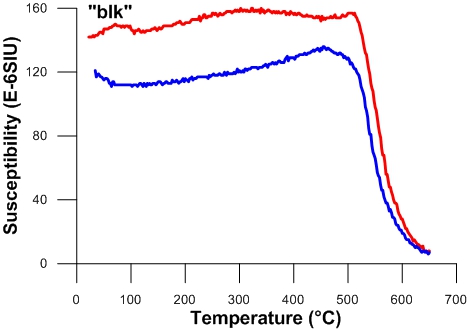

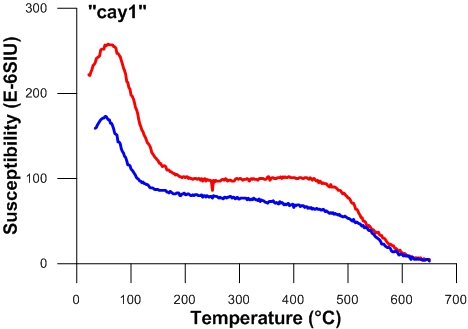


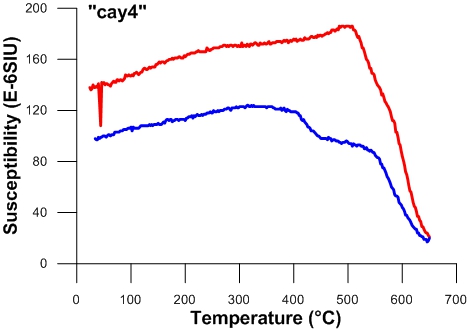

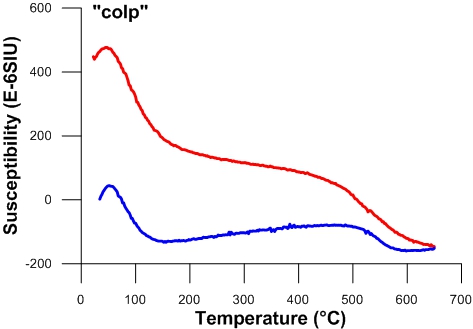


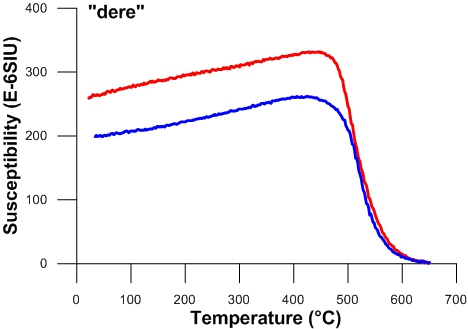

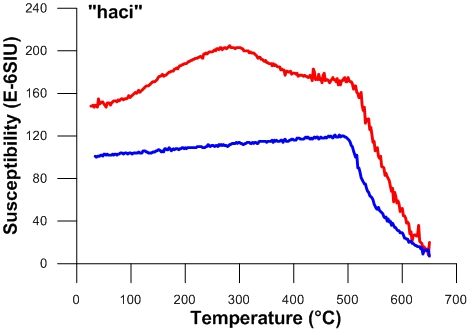


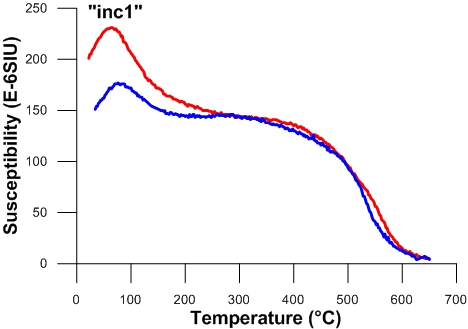

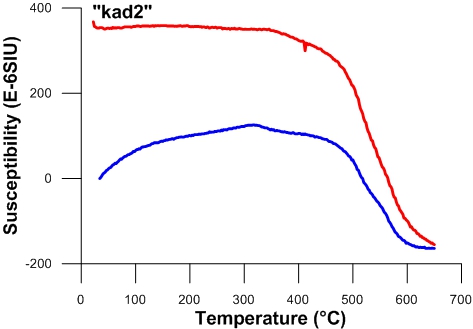


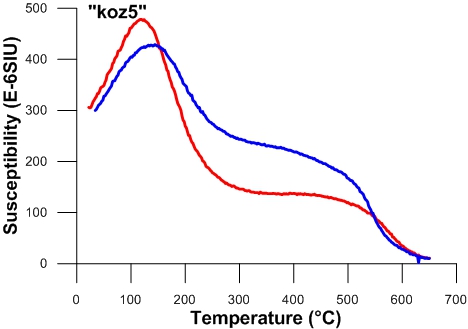

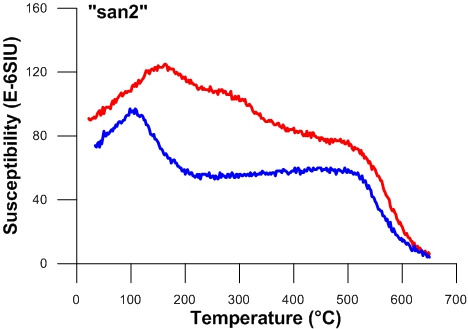


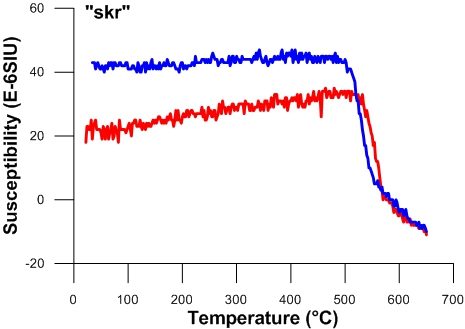

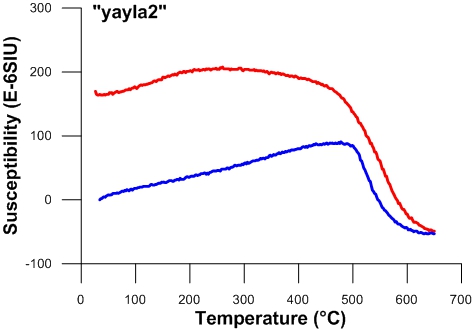


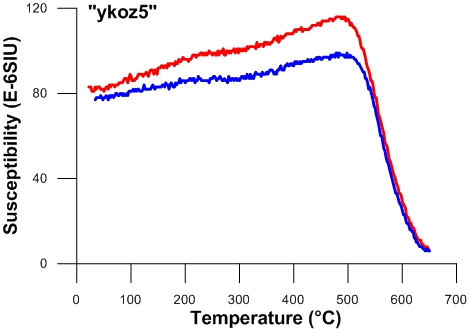

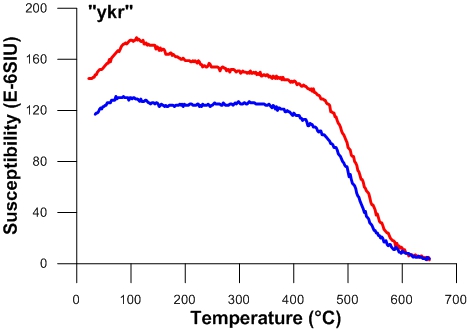

Supplement: Supplementary file 1 — Supplementary Information. [file 41598_2023_39492_MOESM1_ESM.zip › Supplementary_Figure_A2.docx]
